# Revealing the Structure of 6-Aminopenillanic Acid: The Active Nucleus of Penicillins

**DOI:** 10.1021/acs.jpclett.3c03301

**Published:** 2024-02-12

**Authors:** Sergio Mato, Santiago Mata, Elena R. Alonso, Iker León

**Affiliations:** Grupo de Espectrocopía Molecular (GEM), Edificio Quifima, Laboratorios de Espectroscopia y Bioespectroscopia, Unidad Asociada CSIC, Parque Científico UVa, Universidad de Valladolid, 47011 Valladolid, Spain

## Abstract

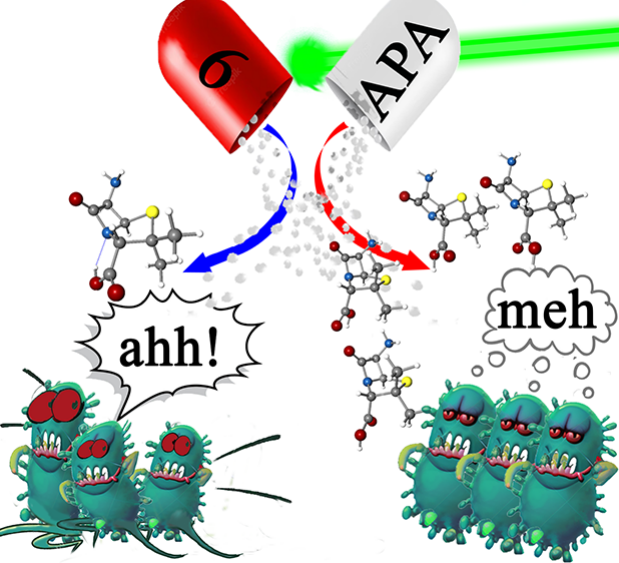

6-Aminopenicillanic acid is a penicillanic acid compound and is the active nucleus common to all penicillins. Using laser ablation techniques, we transformed the solid into the gas phase and characterized its conformational panorama by combining supersonic expansions and Fourier transform microwave techniques. Five conformers were determined, adopting different spatial configurations. Among them, the axial and equatorial forms, which are biologically relevant, have been observed. The structural similarity to d-Ala-d-Ala and the detection of both axial and equatorial forms could explain its potential as a penicillin core and its capability as an antibiotic.

Humans and most other animals carry millions of bacteria. While most of them are suppressed by the immune system and are harmless or beneficial, several classes are pathogenic and cause infectious diseases.^1^ Antibiotic medications are widely used to treat and prevent bacterial infections by killing or inhibiting bacterial growth. One of the most widely used broad-spectrum antimicrobial treatments, such as β-lactam antibiotics, resides in the penicillin family.^2^ They inhibit the catalytic activity of bacterial DD-transpeptidase enzymes, also known as penicillin-binding proteins (PBPs). These enzymes cross-link the peptidoglycan multilayer during cell wall synthesis, resulting in cell death when blocked. The ability of antibiotics to covalently bind with PBPs is due to their structural and stereochemical resemblance to the d-Ala-d-Ala terminus of the peptidoglycan.^2,3^Figure 1 shows penicillin’s scheme, which consists of a side chain and a common nucleus known as 6-aminopenicillanic acid (6-APA). 6-APA is used as an intermediate in the synthesis of β-lactam antibiotics, as it possesses the essential ingredients for most antibiotics. It has a carbonyl in the β-lactam ring, which is a highly reactive point and is responsible for the transient covalent bond with the DD-transpeptidases. Additionally, it has a side chain at the 6-amino position that may be substituted to form semisynthetic penicillins, resulting in various antibacterial and pharmacologic characteristics.

**Figure 1:**
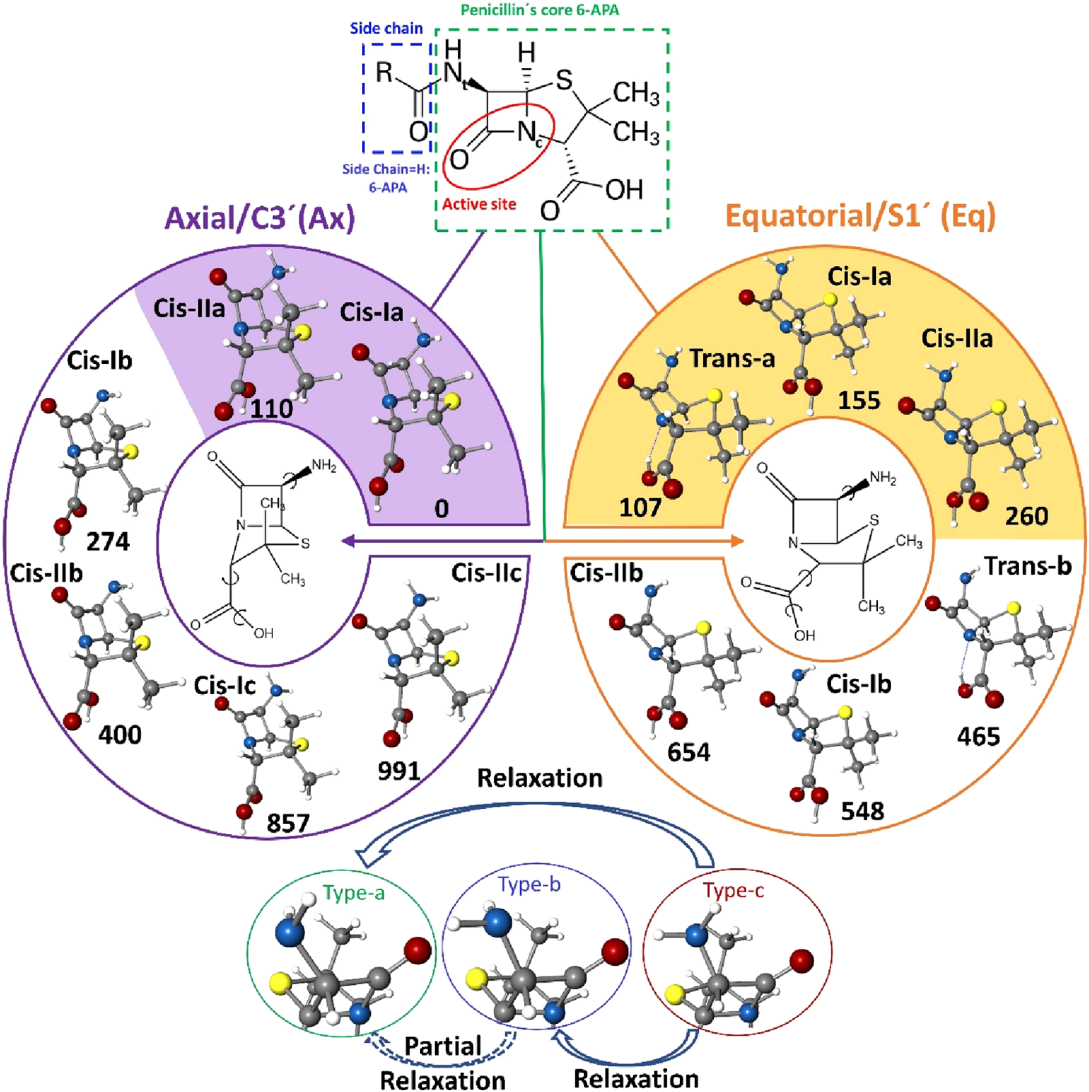
Molecular sketch of penicillin (top). R accounts for a variable group. The core, 6-APA, is also indicated, highlighting the carbonyl group, the leading active site of antibiotics. Relevant most stable conformers of 6-APA (middle). The colored regions are the experimentally detected conformers. Possible relaxation pathways (bottom) (see the text).

Therefore, the structural characterization of 6-APA, the core of penicillin and β-lactams, is mandatory for understanding its mechanisms of action and designing better and more efficient antibiotics. Furthermore, bacterial resistance occurs due to the production of β-lactamases that open the β-lactam ring. Thus, understanding the structure of 6-APA could also lead to new strategies for protecting the ring. Despite the relevance of 6-APA, there is a lack of accurate experimental structural data. Most of the studies published so far were conducted in condensed phases where 6-APA exists in its zwitterionic form. X-ray diffraction and solid-state nuclear magnetic resonance^4−6^ investigations dictated that 6-APA is stabilized by three charge-assisted hydrogen bonds between the ammonium and carboxylate groups, with the thiazolidine ring in an axial configuration. In solution, the results are consistent with a rapid equilibrium between the axial and equatorial forms.^5^ The results using Fourier transform infarred and Fourier transform Raman techniques^7^ provide the vibrational modes of 6-APA, but no structural data are given.

In the context of penicillin properties in solution, an equilibrium between the axial and equatorial forms exists, with the former prevailing.^5^ Cohen proposed that only the less stable equatorial conformation is biologically active.^8^ Along the same line, Mucsi et al. suggested that the ability of penicillins to switch from the anionic to neutral forms of the carboxylic groups acts as a reactive selector of the carbonyl group. When the molecule is at the blood pH (close to pH 7), the antibiotic is in its deprotonated form (COO-).^9^ This form presents an electron repulsion between the carboxylic and nitrogen lone pair, pushing the latter into the amide bond, strengthening the amide bond, and decreasing the reactivity of the carbonyl group. This leads to an inactive form of the antibiotic, maximizing its chance of reaching the receptor without reacting. When the molecule reaches the PBP target, the carbonyl group is protonated, and an intramolecular CO–OH···N group can be formed, which withdraws the density from the amide bond, weakening it and leading to an extremely high reactivity of the carbonyl group. Then, the antibiotic exerts its main action.

To provide meaningful structural information about neutral 6-APA, we performed rotational spectroscopy in combination with supersonic expansions. This technique leads to accurate rotational parameters that can distinguish between different neutral forms unambiguously without any structural perturbations from the environment. It is ideal for studying the inherent structure and properties of 6-APA.

6-APA is a solid with a high melting point (199 °C), and its thermal instability prevents vaporization by classical heating methods. Laser ablation techniques combined with Fourier transform microwave spectroscopy have boosted the investigation of several important biomolecules, allowing their transfer into the gas phase.^10−13^ Following such improvements, we have produced neutral 6-APA using picosecond laser pulses in combination with a chirped-pulse Fourier transform microwave (LA-CP-FTMW)^14,15^ spectrometer. The resulting broadband spectrum between 1.5 and 6.5 GHz is shown in Figure 2 (see also Figure S01). The very dense and complex spectrum anticipates several structures of 6-APA.

**Figure 2:**
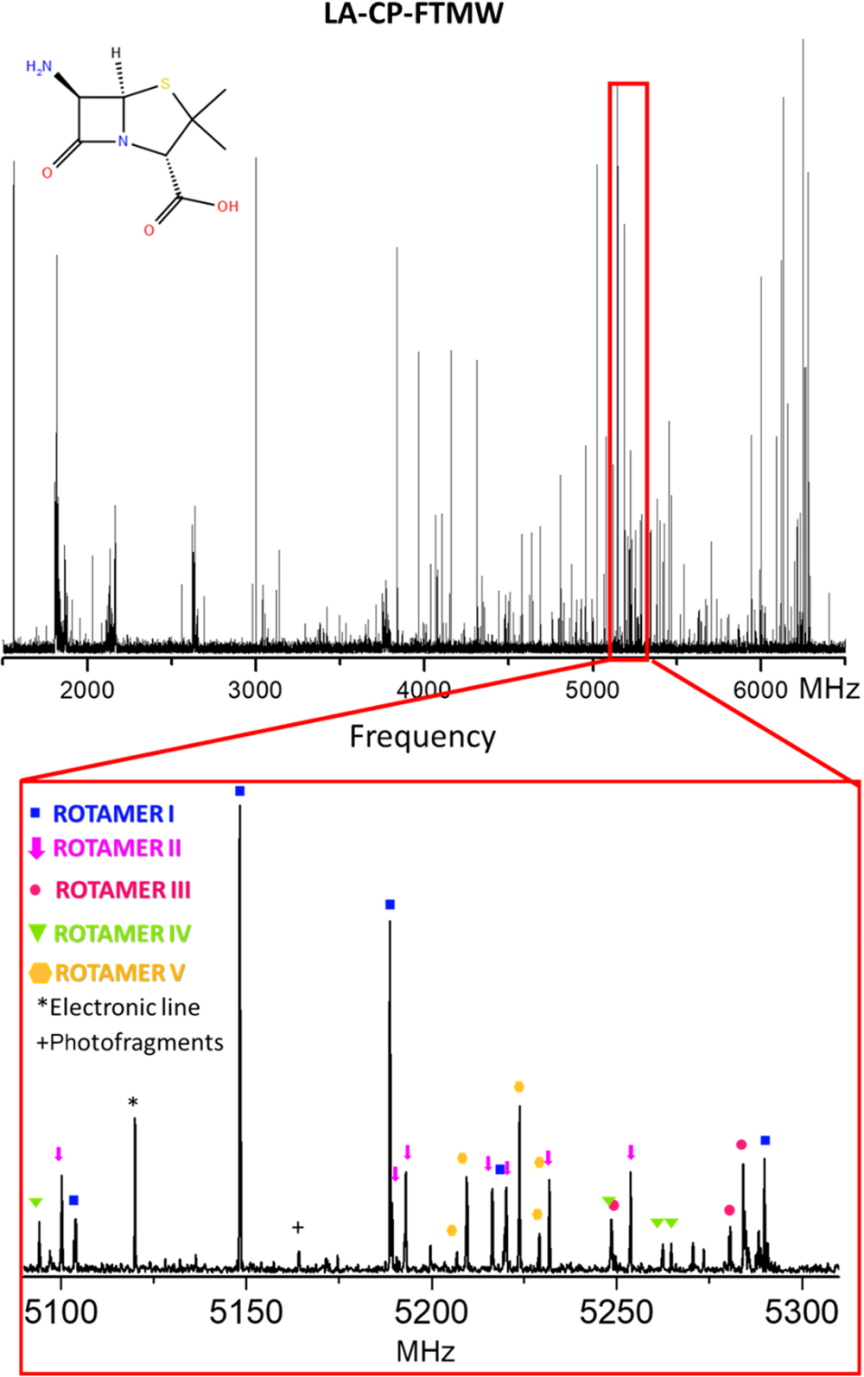
Broadband rotational spectrum of 6-APA in the 1.5–6.5 GHz frequency range (top). Selected spectral section highlighting the transitions of the five detected rotamers (bottom).

To make the conformational search easier, we used a computational strategy that focused on finding and characterizing all of the low-energy minima and their relative stability. The full description can be found in the Supporting Information. In summary, multiple molecular structures were tentatively screened using molecular mechanics, which were subjected to MP2 methods using the 6-311++G(d,p) basis set.^16,17^ Fifteen conformers (Figure S02) were found within an energy window of 2000 cm^–1^ and labeled according to intramolecular interactions or spatial disposition. In Figure 1, the most stable conformations below 1000 cm^–1^ are represented. There are two possible configurations, depending on the configuration of the five-membered thiazolidine ring (penam). These are denoted as pseudoaxial (also known as C-3′), where the β-lactam moiety is folded toward the concave face of the five-membered ring (thiolane), leading to a compact molecule, or pseudoequatorial (S-1′), when it is folded away from it. Additionally, the amino group can point toward the sulfur atom and carbonyl group. We will name this category type a. Structures in type b will comprise those structures in which the amino group points away from the carbonyl group but still interacts with the sulfur atom, similar to type a. The loss of interaction with the carbonyl group slightly destabilized this type of structures. Type c comprises those structures with the amino group pointing away from the bicyclic rings. The structures in this group lose the interaction with the sulfur atom or carbonyl group, which greatly destabilizes them. Finally, the hydroxyl of the carboxylic group can be oriented away (I) or toward the bicyclic ring (II).

We started our experimental conformational search using the predicted values in Table 1 as a starting point. The characteristic pattern of a nearly prolate asymmetric top with sets of a-type R-branch transitions separated approximately by *B + C* was easily observed and ascribed to the first rotamer (I). All of the assigned lines exhibited a poorly resolved hyperfine structure, indicative of a molecule with two ^14^N nuclei (N_c_ and N_t_), each possessing a non-zero electric quadrupole moment (*I* = 1). It arises from the interaction with the electric field gradient created by the rest of the molecule at the nuclei. These observations confirm that the lines correspond to 6-APA. However, only the center of the lines was measured and fitted to a rigid rotor Hamiltonian initially. An iterative procedure of fittings and predictions made identifying b- and c-type transitions possible.

**Table 1:** Experimental Spectroscopic Parameters Obtained for Detected Rotamers I–V of 6-APA Compared with Those Calculated via the Ab Initio MP2/6-311++G(d,p) for the Five Lowest-Energy Conformers (see Tables S01 and S02 for the rest of the calculated conformers)

	experimental					calculated				
	rotamer I^f^	rotamer II^f^	rotamer III	rotamer IV	rotamer V	Ax-*cis*-Ia	Eq-*trans*-a	Ax-*cis*-IIa	Eq-*cis*-Ia	Eq-*cis*-IIa
*A*^a^	961.69106(44)^g^	941.45267(23)	953.6249(41)	954.3031(40)	945.3194(44)	965	947	957	961	952
*B*	532.41954(20)	557.74861(21)	537.68845(222)	541.22403(188)	548.1012(43)	530	555	535	537	544
*C*	485.42941(14)	429.5689(10)	489.69192(291)	429.54997(95)	431.15724(261)	487	432	490	432	434
|*μ*_*a*_|	observed	observed	not observed	observed	not observed	1.9	3.2	0.5	1.5	0.4
|*μ*_*b*_|	observed	observed	observed	observed	observed	0.8	1.8	1.0	1.6	1.0
|*μ*_*c*_|	observed	observed	observed	not observed	observed	0.8	1.7	1.3	0.1	2.2
*χ*_*aa*_/N_r_	1.607(7)	1.322(12)	–	–	–	1.66	1.4	1.62	1.69	1.62
*χ*_*bb*_/N_r_	–2.545(10)	–0.191(13)	–	–	–	–2.55	–0.22	–2.54	–0.43	–0.36
*χ*_*cc*_/N_r_	0.938(10)	–1.131(13)	–	–	–	0.89	–1.17	0.92	–1.26	–1.26
*χ*_*aa*_/N_a_	1.802(12)	2.004(12)	–	–	–	2.08	2.19	2.12	2.20	2.19
*χ*_*bb*_/N_a_	1.484(13)	–1.500(13)	–	–	–	1.53	–1.49	1.54	–1.42	–1.44
*χ*_*cc*_/N_a_	–3.286(13)	–0.504(13)	–	–	–	–3.62	–0.70	–3.66	–0.77	–0.75
σ^b^	3.0	3.4	97.2	57.9	83.3	–	–	–	–	–
*N*^c^	42	42	43	54	31	–	–	–	–	–
Δ*E*_ZPE_^d^	–	–	–	–	–	0	107	110	155	260
Δ*G*^e^	–	–	–	–	–	0	178	98	129	245

a *A, B*, and *C* represent the rotational constants (in megahertz). *μ_a_*, *μ_b_*, and *μ_c_* are the electric dipole moment components (in debye). *χ_aa_*, *χ_bb_*, and *χ_cc_* are the diagonal elements of the ^14^N nuclear quadrupole coupling tensor (in megahertz). N_r_ and N_a_ correspond to the ring and amine ^14^N nuclei, respectively.

b Root-mean-square deviation of the fit (in kilohertz).

c Number of measured transitions.

d Relative energies (in inverse centimeters) with respect to the global minimum, considering the zero-point energy (ZPE).

e Gibbs energies (in inverse centimeters) calculated at 298 K.

f The values have been determined using the LA-MB-FTMW spectrometer.

g Standard error in parentheses in units of the last digit.

Afterward, the lines due to rotamer I were removed from the spectrum to search for other conformers. Following the same strategy, four additional rotamers were located (see the Supporting Information). The obtained rotational constants are listed in Table 1, whereas lists of the measured frequencies can be found in Tables S18–S22.

In the next step, we proceeded with the conformational identification employing various spectroscopy tools, such as rotational constants and selection rules. As our primary criterion, we utilized rotational constants. The axial and equatorial forms can be easily distinguished due to the beta-lactam moiety being folded toward and away from the concave face of the five-membered ring (thiolane), respectively. This difference results in a more compact molecule of the former, resulting in very different values of the *C* rotational constant. Thus, two axial and three equatorial conformers are obtained. Among them, the subtle variation in the mass distribution of the carboxylic hydrogen from *cis* I to II has a minor effect on the value of the *A* rotational constant. Nevertheless, this subtle change dramatically affects the selection rules. Therefore, the predicted dipole moment components can be employed as a second criterion to support identification. Thus, rotamers I and III correspond to conformers Ax-*cis*-I and Ax-*cis*-II, respectively. The same analogy applied to rotamers IV and V sets them as conformers Eq-*cis*-I and Eq-*cis*-II, respectively. The remaining conformer, rotamer II, corresponds to the Eq-*trans* conformer.

However, the distinction between the position of the amino group for some species is not that simple. Tables S01 and S02 show that the Ax-*cis*-Ia and Ax-*cis*-Ib conformers have similar rotational constants and exhibit only small differences in the selection rules, which is insufficient for a conclusive assignment. The same occurs with Eq-*trans*-a and Eq-*trans*-b. Fortunately, the two ^14^N nuclei in the molecule produce a complex hyperfine structure that splits each rotational line in multiple hyperfine components, as illustrated in Figure S03. This is important as it provides information from the electronic environment of the nitrogen nuclei, which is very sensitive to the orientation of the NH_2_ group. We took advantage of the sub-Doppler resolution of the cavity-based LA-MB-FTMW technique^18,19^ to resolve the hyperfine structure of rotamers I and II. A total of 42 hyperfine components were measured for rotamers I and II (Tables S23 and S24). The final values are listed in Table 1. As one can see, despite the quadrupole coupling values for the nitrogen in the ring (N_r_) being similar for both Ax-*cis*-Ia and Ax-*cis*-Ib, those values for the terminal amino group (N_a_) are very different between the conformers (see Tables S01 and S02). All of this information allowed us to unambiguously assign rotamers I–V as conformers Ax-*cis*-Ia, Eq-*trans*-a, Ax-*cis*-IIa, Eq-*cis*-Ia, and Eq-*cis*-IIa, respectively.

With regard to the nondetection of some low-energy conformers, such as structure Ax-*cis*-Ib, a plausible explanation could be a conformational relaxation caused during the supersonic expansion.^20−22^ We performed a relaxed potential energy scan to test it. The results in Figure S04a show that a simple rotation of the amine group interconverts type c structures into type a or b structures through a small barrier of only ∼150 cm^–1^. Type b structures have an interconversion barrier into type a of ∼800 cm^–1^, but the amine inversion lowers the barrier to ∼500 cm^–1^ (see Figure S04b). Additionally, the interconversion path can be more complicated. For example, a concerted motion that involves ring puckering and rotation/inversion of the amino group could lead to a lower barrier. All of these results point to a partial interconversion of type b structures, decreasing the population and precluding their detection.

Once the conformational assignment has been made, we extract some potential biological implications. 6-APA is a convenient starting point for preparing new penicillins by adding different side chains to the amino group. Hence, the fact that the nuclear quadrupole constants allow us to discern the position of NH_2_ is crucial for understanding their synthetic implications. An important fact is that all detected conformers belong to the type a category, where the amino group points inward toward the bicyclic ring, leaving the amino group’s lone pairs susceptible to an electrophilic attack (see Figure S05). It could explain the use of 6-APA as a core to synthesize new structures via modification of its side chain.

Another potential implication of our results lies in the different properties that the axial and equatorial dispositions may offer the molecules and their environment in the context of the structure–activity relationship. Our results can be used to support and combine the hypotheses of both Cohen and Mucsi. First, our results show that the axial and equatorial dispositions are stable and relevant, like what is observed in solution. Furthermore, despite the axial form being slightly more stable, the equatorial form has more conformational variety. Second, we have confirmed the variation of the carbonyl reactivity of the axial and equatorial forms. We analyzed the intramolecular interactions using NCI^23,24^ and QTAIM^25^ (see Figures S06 and S07 and Tables S25 and S26), and the results show that there is a very strong CO–OH···N hydrogen bond in the Eq-*trans* conformer, which has important biological implications. The data in Tables S25 and S26 show that the electronic densities in the N1–C3 bond (β-lactamic bond) of the Ax-*cis*-Ia and Eq-*trans*-a conformers are 0.292 and 0.284, respectively (Δρ ∼ 0.01), meaning that the C3–N1 bond of the Eq-*trans*-a conformer is weaker than that of Ax-*cis*-Ia. One can also see that these data agree with the lower energy densities (V, G, and H) for the same bond calculated for Eq-*trans*-a as for Ax-*cis*-Ia. Therefore, all data indicate that the C3–N1 bond is considerably weaker in the Eq-*trans*-a conformer. This bond weakening comes from the CO–OH···N hydrogen bond, which extracts electronic density from the adjacent C3–N1 pair due to the nitrogen’s lone pair being part of the hydrogen bond, which leads to a smaller contribution of these electron pairs in the N1–C3 bond resulting in a weakening of the bond. Moreover, this explains why the atomic nuclear charge (Table S27) for N1 in Ax-*cis*-Ia is higher than that in Eq-*trans*-a. Altogether, these computational results are consistent with the weaker C3–N1 bond in the Eq-*trans*-a conformer. The fact that a PBP enzyme specializes in attacking this bond means that the enzyme would need less energy to break such a bond; in other words, breaking this bond would be more efficient. This explains why the Eq-*trans* conformer is the most biologically active form. Thus, the carbonyl group is more reactive in the *trans* conformers than in the *cis* forms.

We found only one conformer in a *trans* disposition, and interestingly, conformers in this arrangement have been observed only in the equatorial form. The *trans* disposition in the axial form is not detected experimentally, in good agreement with the calculations that set them as considerably less stable.

The results presented above could imply that the carboxylic group is deprotonated and inactive under the blood pH conditions. Upon protonation, an equilibrium of Ax-*cis*-I, Eq-*trans*, Ax-*cis*-II, Eq-*cis*-I, and Eq-*cis*-II exists, where only Eq-*trans* leads to a highly reactive carbonyl group to bind the protein covalently. This is because the axial *trans* disposition is not biologically accessible as our results suggest. All of these facts support Mucsi’s hypothesis and explain why, as Cohen suggested, only the equatorial form is biologically active. We also note that antimicrobial resistance can utilize the same strategy.

Finally, as previously mentioned, the ability of antibiotics to covalently bind with PBPs is due to their resemblance to the d-Ala-d-Ala terminus of peptidoglycan.^2,3^ We recently studied the Ala-Ala dipeptide using rotational spectroscopy,^26^ making it an ideal situation for comparing both structures. The Ala-Ala dipeptide is a very flexible molecule with >70 conformers in a relative energy window of 2500 cm^–1^. However, only two types of structures predominate, with *cis* and *trans* disposition. Figure 3 shows a comparison between 6-APA and the Ala-Ala dipeptide. As shown, the *cis* and *trans* motifs in both molecules are remarkably similar, confirming the structure–property relationship due to the structural and stereochemical resemblance of antibiotics to the d-Ala-d-Ala terminus of peptidoglycan. Additionally, it is also interesting to see that 6-APA offers the same biological outcome but through a completely different chemical approach. In 6-APA, the two cycles confer rigidity on the molecule and the conformational panorama is limited. However, five conformers of similar stability are accessible in contrast with the two conformers observed in the Ala-Ala dipeptide despite its larger conformational panorama. Thus, the greater spatial floppiness of the Ala-Ala dipeptide can be achieved by the greater conformational diversity of 6-APA. While the Ala-Ala dipeptide can easily adapt to the PBP-binding site, 6-APA could use its diversity to fit the receptor.

**Figure 3:**
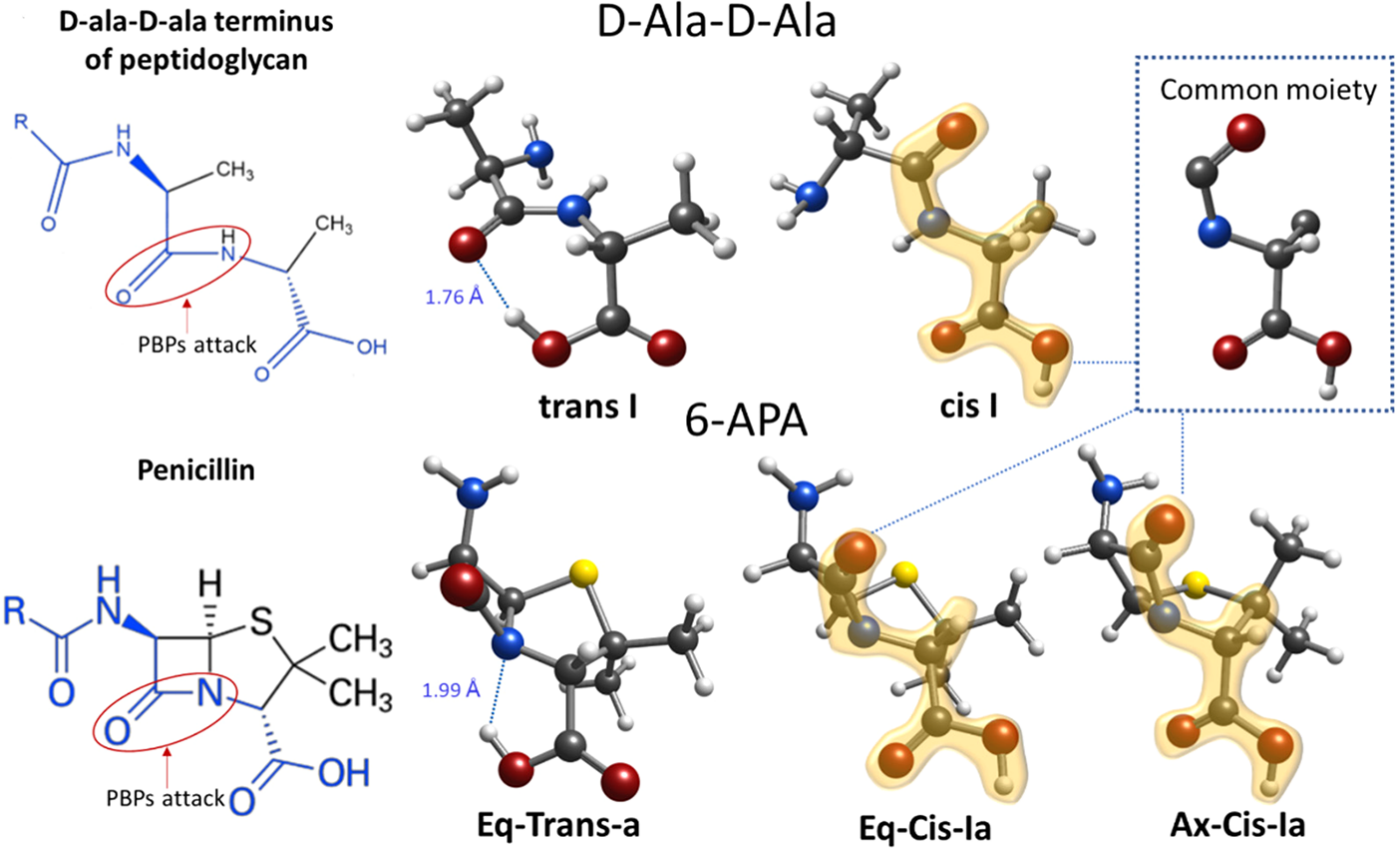
Structural comparison between the most relevant structures detected in the gas phase for the d-Ala-d-Ala terminal dipeptide of peptidoglycan and its structural analogy to 6-APA. The red cycle indicates the point at which the cleavage of the bond occurs in penicillins (active site) to bind to the PBPs.

As a final remark, Figure S08 compares the structure of the most stable species of 6-APA in the gas phase (Ax-*cis*-Ia), with the only conformer observed in the crystal^4^ finding an almost perfect match. Therefore, for APA the predominant conformation in the gas phase matches the molecular spatial disposition in the crystal. The axial configuration allows for better packaging and maximizes the intermolecular interaction between the amine group of a molecule and the carboxylic group of the adjacent molecule.

Antimicrobial resistance is recognized as a major problem in treating microbial infections and is a major cause of death throughout the developing world.^27,28^ We hope our results on 6-APA improve our understanding of the mechanisms of antibiotic resistance and lead to the development of better ways to combat it, such as designing new antibiotics.

## Supporting Information Available

Detailed experimental and theoretical section, detailed view of the configurations of 6-APA, measured frequencies for five rotamers of 6-APA, and topological analysis (PDF)
