# Supplementary material for: Revealing the Structure of 6-Aminopenillanic Acid: The Active Nucleus of Penicillins
Source: J Phys Chem Lett. 2024 Feb 12;15(7):1908–13. doi: 10.1021/acs.jpclett.3c03301 (PMC10895660; doi:10.1021/acs.jpclett.3c03301)
Supplement: Supplementary file 1 — jz3c03301_si_001.pdf [file jz3c03301_si_001.pdf]

Supporting information for:

Revealing the Structure of 6-Aminopenillanic Acid: The Active Nucleus of Penicillins.

S.Mato[a], S. Mata,[a] E. R. Alonso[a], I. León,*[a]

[a]Grupo de Espectroscopía Molecular (GEM), Edificio Quifima, Laboratorios de Espectroscopia y Bioespectroscopia, Unidad Asociada CSIC, Parque Científico UVa, Universidad de Valladolid, 47011 Valladolid, Spain.

*Corresponding Author:

Iker León Ona, iker.leon@uva.es

phones: +34 983 186344 / +34 983 186349

web: http://www.gem.uva.es/

METHODS

Experimental details

A commercial sample of 6-aminopenicillamic acid (Cymit, m.p. 199ºC) was used without prior purification. A solid rod was prepared by pressing a fine compound powder, mixed with a small amount of a commercial binder (Acryl 33), and placed in the ablation nozzle. Next, 6-APA was transferred to the gas phase using a picosecond Nd-YAG laser (17 mJ per pulse, 20 ps pulse width, 355nm). The laser ablation products were then supersonically expanded using a carrier gas flow (Ne, 10 bar) in the vacuum chamber of the spectrometer and characterized by the Fourier transform pulse chirp microwave spectrometer (LA-CP-FTMW). [1,2] The chirp pulse of 4 μs created by the 25 GS s-1 arbitrary waveform generator was amplified by 20 W solid-state amplifier that polarized the molecules in the 1.5-6.5 GHz region and broadcasted across the chamber of the spectrometer through a first microwave horn. At a repetition rate of 2 Hz, a total of 138000 free induction decays (4 FID emissions per gas pulse and 10 μs time of acquisition per FID) were received by a second microwave horn, amplified, and digitized using a 25 GS s^{-1} oscilloscope in the time domain. Then, the broadband spectrum is converted in the frequency domain with Fourier transform. The global spectrum is recorded in Figure 2 of the main text. A second spectrum, shown in Figure S01, was carried out in the 2-8 GHz region using a TWT amplifier with a 300W polarization power averaging 62k FID. The measured rotational transitions in Tables S18-S22 correspond to both spectra.

Figure S01. Experimental spectrum of 6-APA in the region 2-8 GHz employing a TWT amplifier.

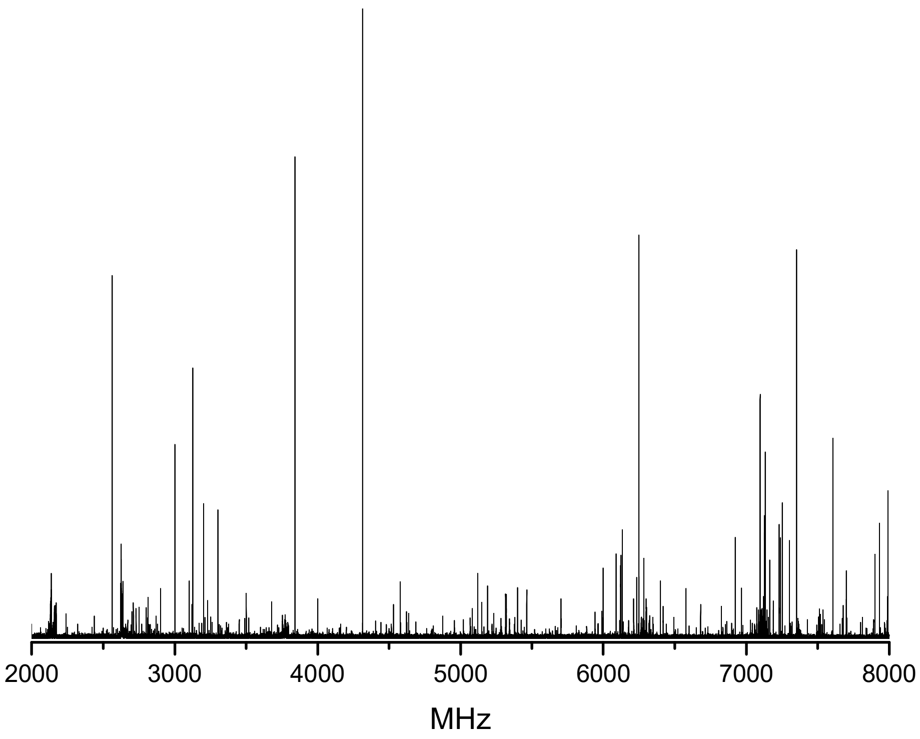

A laser ablation molecular beam Fourier transform microwave (LA-MB-FTMW) spectrometer operating between 2 and 8 GHz was used to resolve the hyperfine structure due to two ^{14}N nuclei. A short microwave pulse with a duration of 0.3 μs and 1 dBm of power was applied to polarize the vaporized molecules. Fourier transform converted the detected free induction decay to the frequency domain. All transitions appear as Doppler doublets due to the coaxial configuration of the molecular beam and microwave radiation. The resonance frequency was determined as an arithmetic mean of the two Doppler components.

Theoretical Modeling

The conformational landscape of 6-APA was explored using a systematic approach to facilitate the identification of the different species present in the supersonic expansion. Initially, we scanned the potential energy hypersurface (PES) using molecular mechanics methods (MMFFs, AMBER and OPLS2005) that implement two search algorithms “Large scales Low Mode” (which uses frequency modes to create new structures) and “Monte Carlo-based search algorithm”, as implemented in Macromodel[3]. A total of 20 conformations were found in an energy window of 50 kJ/mol. Subsequently, the distinctive configurations were optimized with B3LYP-D3(BJ)/6-311++G(d,p) and MP2/6-311++G(d,p) methodology. [4–8] As a result, the initial number of 20 structures decreased to 15. We performed a frequency calculation to verify that these structures are true minima in the PES. These structures are shown in Figure S02. From all these conformers, the predicted spectroscopic parameters, namely rotational constant (*A*, *B* and *C*), quadrupole coupling constants (χ_{aa}, χ_{bb} and χ_{cc}), and electric dipole moment components in the principal inertial axes (μ_a, μ_b and μ_c), as well as their energetics, were extracted and collected in Tables S01 and S02.

Figure S02. Predicted low-energy conformers of 6-APA at MP2/6-311G++(d,p). Black dotted frames highlight the experimentally observed conformers. Relative electronic energies considering the zero-point energy and Gibbs free energies at room temperature are also indicated (ΔE/ΔG). Values are given in cm^{-1}.

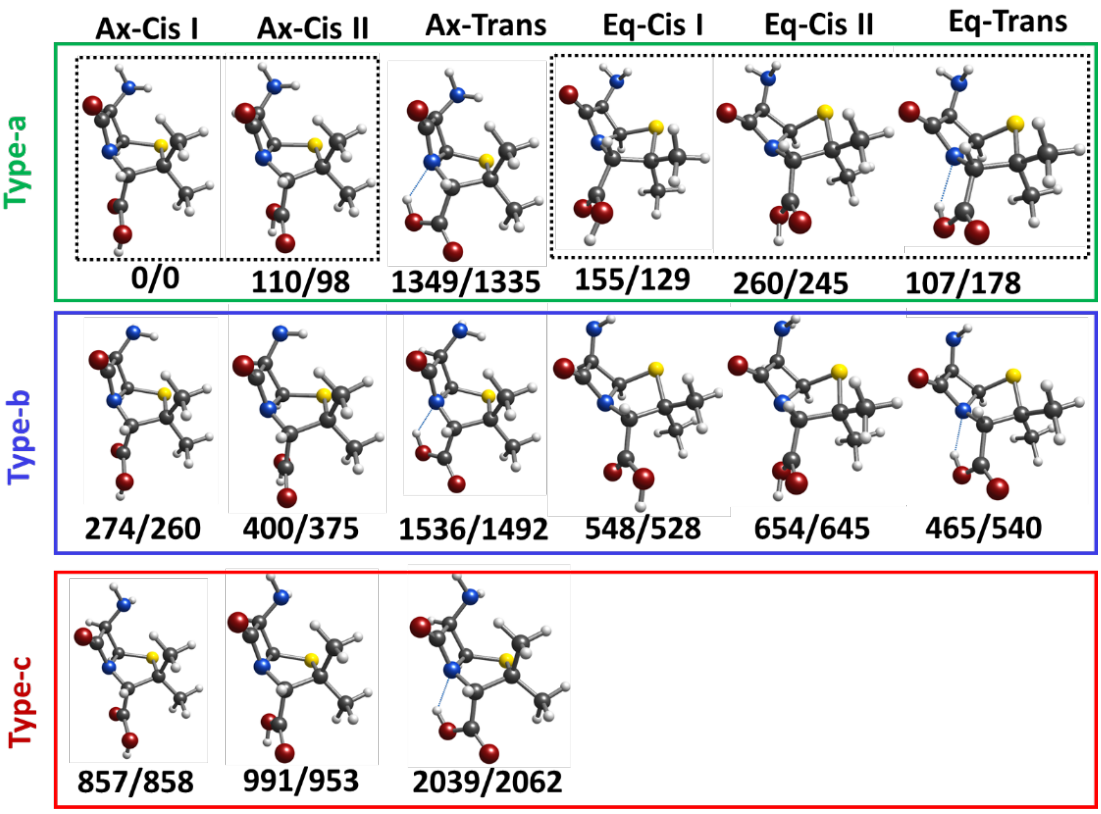

Table S01. Theoretical spectroscopic parameters for the calculated structures of 6-APA at MP2/6-311G++(d,p).

Parameters	Ax-Cis-Ia	Eq-Trans-a	Ax-Cis-IIa	Eq-Cis-Ia	Eq-Cis-IIa	Ax-Cis-Ib	Ax-Cis-IIb	Eq-Trans-b	Eq-Cis-Ib	Eq-Cis-IIb	Ax-Cis-Ic	Ax-Cis-IIc	Ax-Trans-a	Ax-Trans-b	Ax-Trans-c
**A**[a]	965	947	957	961	952	967	959	947	962	952	957	950	930	931	926
**B**	530	555	535	537	544	528	533	553	535	542	547	552	552	553	568
**C**	487	432	490	432	434	485	489	431	431	433	489	493	476	476	481
$\|\mu_a\|$	1.9	3.2	0.5	1.5	0.4	2.4	1.0	2.8	1.9	0.8	0.4	0.8	3.1	2.8	4.5
$\|\mu_b\|$	0.8	1.8	1.0	1.6	1.0	1.8	2.0	3.6	3.3	2.8	1.4	1.5	0.9	1.7	1.2
$\|\mu_c\|$	0.8	1.7	1.3	0.1	2.2	1.1	3.1	2.9	1.3	3.4	0.5	2.6	0.1	1.8	1.5
χ_{aa} / N_r	1.66	1.40	1.62	1.69	1.62	1.65	1.61	1.41	1.71	1.64	1.38	1.34	1.34	1.29	1.11
χ_{bb} / N_r	-2.55	-0.22	-2.54	-0.43	-0.36	-2.62	-2.61	-0.28	-0.51	-0.42	-1.93	-1.99	-2.63	-2.65	-2.27
χ_{cc} / N_r	0.89	-1.17	0.92	-1.26	-1.26	0.97	1.00	-1.13	-1.20	-1.22	0.55	0.65	1.30	1.37	1.17
χ_{aa} / N_a	2.08	2.19	2.12	2.20	2.19	-0.31	-0.35	0.76	0.61	0.60	-0.09	-0.05	1.97	-0.01	-0.05
χ_{bb} / N_a	1.53	-1.49	1.54	-1.42	-1.44	1.07	1.04	-1.82	-1.70	-1.76	1.04	0.98	1.93	1.54	0.87
χ_{cc} / N_a	-3.62	-0.70	-3.66	-0.77	-0.75	-0.76	-0.70	1.06	1.09	1.16	-0.95	-0.93	-3.90	-1.53	-0.82
**ΔE**[b]	0	77	107	151	231	325	450	469	581	663	972	1115	1336	1586	2126
ΔE_{ZPE}[c]	0	107	110	155	260	274	400	465	548	654	857	991	1349	1536	2039
**ΔG**[d]	0	178	98	129	245	260	375	540	528	645	858	953	1335	1492	2062
$BP_{\Delta E}$(%)[e]	27.5%	19.0%	16.4%	13.2%	9.0%	5.7%	3.1%	2.9%	1.7%	1.1%	0.3%	0.1%	0.0%	0.0%	0.0%
BP_{ZPE}(%)	27.8%	16.5%	16.3%	13.1%	7.9%	7.4%	4.0%	2.9%	2.0%	1.2%	0.4%	0.2%	0.0%	0.0%	0.0%
$BP_{\Delta G}$(%)	28.1%	11.9%	17.5%	15.0%	8.6%	8.0%	4.6%	2.1%	2.2%	1.2%	0.4%	0.3%	0.0%	0.0%	0.0%

[a] *A*, *B* and *C* represent the rotation constants (in MHz); μ_a, μ_b and μ_c are the components of the electric dipole moment (in D). χ_{aa}, χ_{bb} y χ_{cc} are the diagonal elements of the ^{14}N nuclear quadrupole coupling tensor in MHz; N_r and N_a correspond to the ring and amine ^{14}N nuclei, respectively.[b] Relative energies (in cm^{-1}) with respect to the global minimum calculated at the level of theory MP2/6-311++G(d,p).[c] Relative energies (in cm^{-1}) with respect to the global minimum, taking into account the zero point energy (ZPE), calculated at the level of theory MP2/6-311++G(d,p).[d] Gibbs energies (in cm^{-1}) calculated at 298 K at the level of theory MP2/6-311++G(d,p).[e] Calculated populations in % assuming a Boltzmann distribution (BP = Boltzmann population) at 298 K and using the $\Delta E/\Delta E_{ZPE}/\Delta G$ values.

Table S02. Theoretical spectroscopic parameters for the calculated structures of 6-APA at B3LYP-D3(BJ)/6-311G++(d,p).

Parameters	Ax-Cis-Ia	Ax-Cis-IIa	Ax-Cis-Ib	Eq-Trans-a	Eq-Cis-Ia	Ax-Cis-IIb	Eq-Cis-IIa	Eq-Trans-b	Eq-Cis-Ib	Eq-Cis-IIb	Ax-Cis-Ic	Ax-Cis-IIc	Ax-Trans-a	Ax-Trans-b	Ax-Trans-c
**A**[a]	948	940	950	924	936	942	927	924	936	927	938	931	914	915	908
**B**	530	535	528	559	541	533	548	557	538	546	548	553	554	553	570
**C**	480	484	478	425	425	482	427	424	425	426	482	486	470	469	475
$\|\mu_a\|$	2.0	0.5	2.3	3.1	1.7	0.9	0.4	2.8	2.0	0.7	0.5	0.8	3.1	2.9	4.5
$\|\mu_b\|$	1.1	1.2	2.0	2.1	2.0	2.1	1.3	3.8	3.6	2.9	1.7	1.8	1.0	1.8	1.5
$\|\mu_c\|$	0.8	1.5	1.0	1.9	0.0	3.3	2.4	3.1	1.2	3.5	0.4	2.8	0.1	1.9	1.7
χ_{aa} / N_r	1.80	1.75	1.81	1.49	1.81	1.75	1.72	1.51	1.83	1.75	1.53	1.48	1.46	1.44	1.21
χ_{bb} / N_r	-2.63	-2.67	-2.70	-0.16	-0.34	-2.74	-0.29	-0.24	-0.44	-0.39	-2.01	-2.16	-2.75	-2.81	-2.33
χ_{cc} / N_r	0.83	0.92	0.90	-1.34	-1.47	0.99	-1.43	-1.27	-1.40	-1.36	0.48	0.68	1.30	1.37	1.12
χ_{aa} / N_a	2.10	2.12	-0.08	2.19	2.16	-0.13	2.15	0.90	0.75	0.72	-0.12	-0.10	2.00	0.24	0.05
χ_{bb} / N_a	1.66	1.72	1.12	-1.51	-1.43	1.16	-1.41	-1.94	-1.86	-1.89	1.52	1.43	2.08	1.59	1.60
χ_{cc} / N_a	-3.76	-3.83	-1.04	-0.68	-0.73	-1.03	-0.74	1.04	1.11	1.17	-1.40	-1.32	-4.08	-1.84	-1.66
**ΔE**[b]	0	127	311	273	359	460	427	649	767	839	1079	1193	1119	1359	2013
ΔE_{ZPE}[c]	0	125	263	300	358	410	438	630	717	800	957	1068	1116	1314	1907
**ΔG**[d]	0	98	252	348	297	371	372	682	659	735	901	987	1114	1299	1874
$BP_{\Delta E}$(%)[e]	39.3%	21.3%	8.8%	10.5%	6.9%	4.3%	5.0%	1.7%	1.0%	0.7%	0.2%	0.1%	0.2%	0.1%	0.0%
BP_{ZPE}(%)	38.2%	20.9%	10.7%	9.0%	6.8%	5.3%	4.6%	1.8%	1.2%	0.8%	0.4%	0.2%	0.2%	0.1%	0.0%
$BP_{\Delta G}$(%)	35.6%	22.2%	10.6%	6.6%	8.5%	5.9%	5.9%	1.3%	1.5%	1.0%	0.5%	0.3%	0.2%	0.1%	0.0%

[a] *A*, *B* and *C* represent the rotation constants (in MHz); μ_a, μ_b and μ_c are the components of the electric dipole moment (in D). χ_{aa}, χ_{bb} y χ_{cc} are the diagonal elements of the ^{14}N nuclear quadrupole coupling tensor in MHz; N_r and N_a correspond to the ring and amine ^{14}N nuclei, respectively.[b] Relative energies (in cm^{-1}) with respect to the global minimum calculated at the level of theory MP2/6-311++G(d,p).[c] Relative energies (in cm^{-1}) with respect to the global minimum, taking into account the zero point energy (ZPE), calculated at the level of theory MP2/6-311++G(d,p).[d] Gibbs energies (in cm^{-1}) calculated at 298 K at the level of theory MP2/6-311++G(d,p).[e] Calculated populations in % assuming a Boltzmann distribution (BP = Boltzmann population) at 298 K and using the $\Delta E/\Delta E_{ZPE}/\Delta G$ values.

Table S03. Cartesian coordinates in Angstroms of 6-APA, *Ax-Cis-Ia*, from the optimized ab initio structure at the MP2/6-311G++(d,p) and B3LYP-D3(BJ)/6-311G++(d,p) levels.

MP2/6-311G++(d,p)			
Atom	X	Y	Z
N	-0.370615	1.049848	-0.031008
C	-0.96308	0.387721	1.139725
C	-1.597691	1.089155	-0.721316
C	0.855603	0.471829	-0.533394
S	-0.380214	-1.344416	1.074883
C	0.726599	-1.083122	-0.388293
C	0.07613	-1.652967	-1.653844
C	2.060309	-1.78651	-0.123779
C	-2.376702	0.706048	0.566695
O	-1.856422	1.281026	-1.885116
N	-3.343816	-0.360073	0.555726
C	2.025946	1.023399	0.256851
O	3.134578	1.10934	-0.515928
O	1.991189	1.325356	1.426979
H	-0.694732	0.825812	2.100967
H	0.966571	0.733409	-1.590525
H	-0.142216	-2.716352	-1.514998
H	0.760623	-1.546368	-2.504767
H	-0.856637	-1.137264	-1.894908
H	1.896786	-2.865052	-0.034275
H	2.522027	-1.436472	0.803425
H	2.747852	-1.613972	-0.958829
H	-2.806143	1.588666	1.0517
H	-2.966209	-1.175938	0.081874
H	-4.184762	-0.068417	0.065948
H	3.84384	1.435102	0.059872

B3LYP-D3(BJ)/6-311G++(d,p)			
Atom	X	Y	Z
N	-0.383407	1.021975	0.014028
C	-0.984844	0.328863	1.158746
C	-1.595709	1.149769	-0.672797
C	0.851485	0.48136	-0.505105
S	-0.395233	-1.414798	1.04014
C	0.741522	-1.087954	-0.420898
C	0.100126	-1.615617	-1.709681
C	2.074632	-1.79352	-0.163347
C	-2.394408	0.67882	0.582867
O	-1.839148	1.43135	-1.814913
N	-3.350479	-0.388786	0.482558
C	2.026364	1.032426	0.286931
O	3.13101	1.133463	-0.48713
O	2.004038	1.330046	1.451388
H	-0.722322	0.730663	2.133194
H	0.956958	0.780167	-1.548328
H	-0.114968	-2.681515	-1.614874
H	0.785717	-1.477223	-2.552232
H	-0.830757	-1.098161	-1.942141
H	1.919971	-2.872927	-0.107787
H	2.527825	-1.471636	0.775033
H	2.771461	-1.594867	-0.981717
H	-2.839549	1.524314	1.112721
H	-2.94968	-1.200053	0.023125
H	-4.17773	-0.096045	-0.024993
H	3.851808	1.457955	0.074599

Table S04. Cartesian coordinates in Angstroms of 6-APA, *Ax-Cis-IIa*, from the optimized ab initio structure at the MP2/6-311G++(d,p) and B3LYP-D3(BJ)/6-311G++(d,p) levels.

MP2/6-311G++(d,p)			
Atom	X	Y	Z
N	-0.354056	1.04503	-0.076608
C	-0.932579	0.420434	1.122567
C	-1.593441	1.060841	-0.7503
C	0.855669	0.441665	-0.59372
S	-0.359962	-1.315188	1.106117
C	0.738107	-1.105333	-0.37262
C	0.081475	-1.736283	-1.605905
C	2.083299	-1.779505	-0.092206
C	-2.353708	0.724705	0.560774
O	-1.867421	1.207727	-1.916616
N	-3.324994	-0.336403	0.602529
C	2.097871	1.030456	0.044559
O	1.91751	1.289518	1.359968
O	3.138667	1.21762	-0.543582
H	-0.651972	0.889418	2.065685
H	0.920124	0.648612	-1.666592
H	-0.130497	-2.793364	-1.417809
H	0.758972	-1.664342	-2.465794
H	-0.856103	-1.235862	-1.861616
H	1.939056	-2.857587	0.029459
H	2.542977	-1.393531	0.821229
H	2.76641	-1.618406	-0.93361
H	-2.771537	1.625601	1.021901
H	-2.959955	-1.168938	0.148203
H	-4.174155	-0.056988	0.11995
H	2.759212	1.64721	1.682623

B3LYP-D3(BJ)/6-311G++(d,p)			
Atom	X	Y	Z
N	-0.368363	1.019407	-0.037016
C	-0.951666	0.369815	1.143546
C	-1.595128	1.118879	-0.707021
C	0.849964	0.451796	-0.572366
S	-0.357461	-1.373734	1.093621
C	0.749553	-1.111248	-0.403576
C	2.093839	-1.792944	-0.142882
C	0.08383	-1.705868	-1.650562
C	-2.371355	0.693569	0.577434
O	-1.858159	1.354824	-1.854471
N	-3.324134	-0.380821	0.53287
C	2.097807	1.041561	0.064442
O	1.930288	1.334426	1.371497
O	3.135604	1.208089	-0.522383
H	-0.680226	0.814085	2.09724
H	0.907783	0.691852	-1.634079
H	1.952964	-2.87132	-0.045975
H	2.560567	-1.431428	0.773909
H	2.775653	-1.61375	-0.978124
H	-0.125603	-2.766073	-1.497529
H	0.751991	-1.608043	-2.512264
H	-0.853484	-1.202886	-1.889726
H	-2.812179	1.556524	1.082327
H	-2.925881	-1.208769	0.101775
H	-4.158754	-0.111993	0.024065
H	2.771091	1.689362	1.698237

Table S05. Cartesian coordinates in Angstroms of 6-APA, *Eq-Trans-a*, from the optimized ab initio structure at the MP2/6-311G++(d,p) and B3LYP-D3(BJ)/6-311G++(d,p) levels.

MP2/6-311G++(d,p)			
Atom	X	Y	Z
N	-0.386074	0.833523	0.365795
C	-1.193407	-0.257569	0.984364
C	-1.535012	1.296827	-0.351049
C	0.791753	0.394392	-0.392855
S	-0.779771	-1.737329	0.021883
C	0.957387	-1.133666	-0.143418
C	1.731489	-1.419548	1.147981
C	1.620079	-1.8212	-1.334714
C	-2.492638	0.471255	0.542036
O	-1.612291	1.962068	-1.348712
N	-3.552813	-0.316583	-0.02197
C	2.055206	1.165237	-0.006177
O	1.924961	1.970881	1.063035
O	3.094671	1.034312	-0.607254
H	-1.029536	-0.396703	2.054223
H	0.616122	0.575201	-1.460838
H	2.771763	-1.091432	1.046963
H	1.722963	-2.494181	1.348834
H	1.291751	-0.90821	2.010832
H	1.715969	-2.895412	-1.147303
H	2.61977	-1.401359	-1.482222
H	1.034567	-1.671138	-2.246826
H	-2.880053	1.082932	1.363036
H	-3.278058	-0.707474	-0.917988
H	-4.386137	0.244311	-0.164508
H	0.981551	1.929693	1.305321

B3LYP-D3(BJ)/6-311G++(d,p)			
Atom	X	Y	Z
N	-0.410806	0.801624	0.350278
C	-1.206319	-0.285303	0.982852
C	-1.551575	1.314307	-0.32122
C	0.78808	0.388317	-0.389738
S	-0.753795	-1.791328	0.050571
C	0.992691	-1.143615	-0.147649
C	1.787252	-1.429342	1.132897
C	1.646094	-1.812261	-1.354439
C	-2.512017	0.435463	0.530419
O	-1.633121	2.041764	-1.266387
N	-3.537403	-0.352323	-0.090174
C	2.02985	1.201009	-0.006258
O	1.860623	2.055667	1.015263
O	3.081365	1.070253	-0.572039
H	-1.045958	-0.405441	2.052263
H	0.617657	0.567549	-1.454173
H	2.815464	-1.075055	1.028494
H	1.810017	-2.503153	1.320406
H	1.346666	-0.944527	2.00688
H	1.756252	-2.88557	-1.185158
H	2.638104	-1.383212	-1.509768
H	1.05406	-1.660047	-2.258922
H	-2.940156	1.009556	1.355044
H	-3.220603	-0.764292	-0.961005
H	-4.37038	0.194133	-0.272504
H	0.921774	2.013281	1.272705

Table S06. Cartesian coordinates in Angstroms of 6-APA, *Eq-Cis-Ia*, from the optimized ab initio structure at the MP2/6-311G++(d,p) and B3LYP-D3(BJ)/6-311G++(d,p) levels.

MP2/6-311G++(d,p)			
Atom	X	Y	Z
N	-0.421797	0.851985	0.363422
C	-1.214841	-0.214899	1.004563
C	-1.54708	1.325626	-0.341364
C	0.780256	0.439166	-0.335294
S	-0.833152	-1.701496	0.028442
C	0.905767	-1.118573	-0.184712
C	1.727907	-1.507786	1.049173
C	1.492303	-1.75091	-1.446002
C	-2.511974	0.526896	0.57368
O	-1.63269	1.991008	-1.343619
N	-3.587795	-0.253548	0.023465
C	2.009433	1.143737	0.210824
O	3.026861	1.063978	-0.687182
O	2.104751	1.677457	1.288643
H	-1.028915	-0.359254	2.070199
H	0.675207	0.697034	-1.3971
H	1.689046	-2.591961	1.184426
H	1.3487	-1.029632	1.957644
H	2.776493	-1.214698	0.919243
H	1.552629	-2.838197	-1.333146
H	2.503052	-1.365695	-1.614164
H	0.875226	-1.52074	-2.319571
H	-2.883339	1.147566	1.394513
H	-3.32414	-0.645324	-0.87566
H	-4.41101	0.322639	-0.118385
H	3.782633	1.514045	-0.279216

B3LYP-D3(BJ)/6-311G++(d,p)			
Atom	X	Y	Z
N	0.447151	0.814972	-0.345423
C	1.233365	-0.242763	-0.998678
C	1.560551	1.355479	0.306021
C	-0.773191	0.429163	0.332652
S	0.81131	-1.759272	-0.054961
C	-0.937135	-1.133504	0.187619
C	-1.779196	-1.51948	-1.03496
C	-1.513959	-1.749321	1.461251
C	2.533938	0.49832	-0.560218
O	1.644033	2.090977	1.25066
N	3.57602	-0.276194	0.054647
C	-1.984364	1.175498	-0.208414
O	-3.008945	1.113435	0.678966
O	-2.063366	1.732661	-1.268152
H	1.053013	-0.368704	-2.064399
H	-0.673901	0.683551	1.391063
H	-1.768019	-2.602432	-1.160998
H	-1.40188	-1.06274	-1.951161
H	-2.818366	-1.20393	-0.901959
H	-1.587213	-2.834689	1.363481
H	-2.517633	-1.356028	1.639072
H	-0.89064	-1.51955	2.327187
H	2.946636	1.077014	-1.388702
H	3.26614	-0.690997	0.926844
H	4.39388	0.291307	0.242587
H	-3.758987	1.58717	0.28834

Table S07. Cartesian coordinates in Angstroms of 6-APA, *Eq-Cis-IIa*, from the optimized ab initio structure at the MP2/6-311G++(d,p) and B3LYP-D3(BJ)/6-311G++(d,p) levels.

MP2/6-311G++(d,p)			
Atom	X	Y	Z
N	-0.400897	0.844972	0.339151
C	-1.191842	-0.214749	1.00015
C	-1.536787	1.320845	-0.34928
C	0.77936	0.410531	-0.389201
S	-0.822146	-1.715541	0.041719
C	0.915912	-1.136619	-0.18108
C	1.735397	-1.478971	1.068528
C	1.520798	-1.799837	-1.416725
C	-2.491902	0.526634	0.579202
O	-1.63425	1.983654	-1.351922
N	-3.573	-0.256339	0.04255
C	2.063433	1.11504	0.00356
O	2.012759	1.669758	1.231734
O	3.04456	1.136711	-0.708448
H	-0.995012	-0.34626	2.065657
H	0.630375	0.617691	-1.456629
H	1.705512	-2.558857	1.236654
H	1.347242	-0.977753	1.960306
H	2.782054	-1.181556	0.931891
H	1.607428	-2.880497	-1.265106
H	2.522184	-1.393504	-1.593878
H	0.90346	-1.613959	-2.300493
H	-2.85639	1.150349	1.400904
H	-3.315368	-0.657227	-0.854389
H	-4.395198	0.321117	-0.100649
H	2.884562	2.070453	1.374491

B3LYP-D3(BJ)/6-311G++(d,p)			
Atom	X	Y	Z
N	-0.42683	0.808518	0.317009
C	-1.208131	-0.241377	0.994775
C	-1.551785	1.345862	-0.319834
C	0.773136	0.399995	-0.391408
S	-0.79651	-1.773834	0.074489
C	0.950344	-1.150872	-0.181055
C	1.787495	-1.47913	1.061881
C	1.547462	-1.80636	-1.424249
C	-2.513357	0.495111	0.564867
O	-1.649101	2.075371	-1.267466
N	-3.560324	-0.286312	-0.033341
C	2.038328	1.147488	-0.002313
O	1.961359	1.759362	1.194436
O	3.027288	1.163543	-0.692474
H	-1.015936	-0.349691	2.060561
H	0.629618	0.600107	-1.456112
H	2.823171	-1.155594	0.922248
H	1.788869	-2.556575	1.228684
H	1.396012	-0.994824	1.957971
H	1.646372	-2.88451	-1.280272
H	2.541463	-1.39361	-1.612688
H	0.924342	-1.627166	-2.302216
H	-2.919617	1.079493	1.392668
H	-3.255959	-0.712927	-0.901857
H	-4.378058	0.280372	-0.224604
H	2.816655	2.190314	1.346163

Table S08. Cartesian coordinates in Angstroms of 6-APA, *Ax-Cis-Ib*, from the optimized ab initio structure at the MP2/6-311G++(d,p) and B3LYP-D3(BJ)/6-311G++(d,p) levels.

MP2/6-311G++(d,p)			
Atom	X	Y	Z
N	-0.368687	1.052689	-0.02068
C	-0.962473	0.383408	1.141797
C	-1.591674	1.084092	-0.725837
C	0.855641	0.473341	-0.525407
S	-0.380352	-1.345753	1.074526
C	0.726346	-1.083381	-0.388802
C	2.05945	-1.788539	-0.12561
C	0.076707	-1.649276	-1.656433
C	-2.387361	0.70079	0.546212
O	-1.831692	1.288609	-1.888399
N	-3.409828	-0.28842	0.373817
C	2.028236	1.019016	0.265807
O	3.135675	1.107289	-0.508583
O	1.996812	1.314982	1.437584
H	-0.699879	0.821388	2.105806
H	0.966895	0.738816	-1.581624
H	2.520173	-1.443532	0.803976
H	2.74791	-1.611071	-0.958844
H	1.895849	-2.867589	-0.042929
H	-0.14186	-2.713047	-1.521032
H	0.763252	-1.540621	-2.505383
H	-0.855305	-1.133162	-1.898968
H	-2.809733	1.58938	1.026907
H	-3.927658	-0.43492	1.234058
H	-3.006166	-1.177298	0.091123
H	3.846166	1.430072	0.06736

B3LYP-D3(BJ)/6-311G++(d,p)			
Atom	X	Y	Z
N	-0.38054	1.023507	0.02309
C	-0.984477	0.32454	1.158381
C	-1.587499	1.144886	-0.681346
C	0.853419	0.483152	-0.496265
S	-0.39903	-1.416944	1.036072
C	0.741792	-1.08817	-0.422212
C	0.102429	-1.609465	-1.714333
C	2.073069	-1.796762	-0.164486
C	-2.405442	0.674005	0.55578
O	-1.809468	1.443173	-1.820253
N	-3.411055	-0.314019	0.315881
C	2.029418	1.02704	0.299248
O	3.135063	1.127058	-0.473644
O	2.007979	1.320359	1.46487
H	-0.727626	0.725454	2.13581
H	0.96155	0.786544	-1.537972
H	-0.111666	-2.676084	-1.625453
H	0.789981	-1.466419	-2.554459
H	-0.828401	-1.092343	-1.947076
H	1.917223	-2.876318	-0.115871
H	2.52435	-1.48052	0.776742
H	2.772151	-1.59397	-0.979882
H	-2.844859	1.526223	1.078945
H	-3.98333	-0.479944	1.134807
H	-3.005004	-1.195395	0.018342
H	3.856426	1.447388	0.08973

Table S09. Cartesian coordinates in Angstroms of 6-APA, *Ax-Cis-IIb*, from the optimized ab initio structure at the MP2/6-311G++(d,p) and B3LYP-D3(BJ)/6-311G++(d,p) levels.

MP2/6-311G++(d,p)			
Atom	X	Y	Z
N	-0.35258	1.048009	-0.067754
C	-0.931513	0.418821	1.125165
C	-1.588386	1.05499	-0.755078
C	0.854862	0.442906	-0.586989
S	-0.356574	-1.313197	1.11119
C	0.736868	-1.106002	-0.3715
C	2.081634	-1.782265	-0.0948
C	0.077084	-1.734734	-1.6041
C	-2.365053	0.718679	0.540694
O	-1.844527	1.212928	-1.92099
N	-3.392262	-0.272535	0.417924
C	2.100127	1.026356	0.050159
O	1.923436	1.28785	1.36591
O	3.141348	1.207921	-0.539036
H	-0.656668	0.890507	2.069814
H	0.918847	0.651903	-1.659577
H	1.936915	-2.860756	0.022546
H	2.543473	-1.39982	0.819061
H	2.763463	-1.618232	-0.936643
H	-0.135294	-2.79189	-1.416977
H	0.754415	-1.662632	-2.464074
H	-0.85995	-1.233663	-1.859893
H	-2.77792	1.624668	0.996608
H	-3.895386	-0.391167	1.291048
H	-2.99637	-1.170691	0.154357
H	2.767743	1.642518	1.685108

B3LYP-D3(BJ)/6-311G++(d,p)			
Atom	X	Y	Z
N	-0.365526	1.020802	-0.028316
C	-0.951762	0.366021	1.143365
C	-1.586871	1.114152	-0.715882
C	0.851944	0.453132	-0.563585
S	-0.360796	-1.375222	1.092252
C	0.749379	-1.112061	-0.403452
C	2.091804	-1.797074	-0.142539
C	0.084418	-1.701164	-1.653175
C	-2.383229	0.687323	0.55003
O	-1.828165	1.367565	-1.861098
N	-3.386953	-0.313479	0.362102
C	2.101614	1.036663	0.07558
O	1.934014	1.334003	1.381603
O	3.142037	1.194407	-0.509169
H	-0.686614	0.810745	2.099828
H	0.911901	0.69688	-1.624444
H	1.949307	-2.875681	-0.051091
H	2.557397	-1.440183	0.776681
H	2.775545	-1.614685	-0.975477
H	-0.124633	-2.762128	-1.505064
H	0.753917	-1.599501	-2.513341
H	-0.852867	-1.198236	-1.892205
H	-2.819591	1.556112	1.048031
H	-3.94611	-0.452629	1.194905
H	-2.981818	-1.203211	0.089365
H	2.776791	1.685035	1.707539

Table S10. Cartesian coordinates in Angstroms of 6-APA, *Eq-Trans-b*, from the optimized ab initio structure at the MP2/6-311G++(d,p) and B3LYP-D3(BJ)/6-311G++(d,p) levels.

MP2/6-311G++(d,p)			
Atom	X	Y	Z
N	-0.389166	0.82798	0.36806
C	-1.190128	-0.265243	0.984291
C	-1.538393	1.285479	-0.357162
C	0.79064	0.394032	-0.389711
S	-0.77213	-1.741788	0.022714
C	0.964157	-1.134797	-0.144163
C	1.740006	-1.421375	1.14585
C	1.627258	-1.816935	-1.338191
C	-2.500671	0.473902	0.532613
O	-1.607683	1.954095	-1.350274
N	-3.600241	-0.15705	-0.128201
C	2.051262	1.168862	-0.000435
O	1.913444	1.979288	1.06373
O	3.094092	1.036266	-0.595394
H	-1.025206	-0.401409	2.055154
H	0.615618	0.576216	-1.457544
H	2.779386	-1.091289	1.043059
H	1.733971	-2.496267	1.34552
H	1.30049	-0.911225	2.009435
H	1.730042	-2.8909	-1.153163
H	2.624049	-1.390675	-1.486716
H	1.039088	-1.668006	-2.248738
H	-2.869371	1.094468	1.356672
H	-4.192272	-0.667719	0.517106
H	-3.287734	-0.779558	-0.866268
H	0.968275	1.93771	1.300002

B3LYP-D3(BJ)/6-311G++(d,p)			
Atom	X	Y	Z
N	-0.411728	0.799103	0.351059
C	-1.20234	-0.286114	0.984605
C	-1.550635	1.30546	-0.33338
C	0.788866	0.387873	-0.386072
S	-0.751471	-1.791751	0.056285
C	0.995893	-1.145942	-0.150317
C	1.796001	-1.43719	1.125247
C	1.642407	-1.809866	-1.363154
C	-2.519333	0.444728	0.515202
O	-1.620957	2.036381	-1.273411
N	-3.580352	-0.200721	-0.187006
C	2.029891	1.199969	0.002716
O	1.853638	2.060855	1.017246
O	3.085563	1.0635	-0.55404
H	-1.041965	-0.400276	2.055475
H	0.621538	0.571093	-1.450351
H	2.824109	-1.084404	1.015963
H	1.817915	-2.511573	1.309647
H	1.360496	-0.95351	2.002359
H	1.755865	-2.883522	-1.198399
H	2.632703	-1.378245	-1.522394
H	1.044918	-1.655166	-2.263568
H	-2.930302	1.032822	1.339671
H	-4.213839	-0.698744	0.42507
H	-3.246148	-0.819443	-0.916837
H	0.912362	2.020903	1.266986

Table S11. Cartesian coordinates in Angstroms of 6-APA, *Eq-Cis-Ib*, from the optimized ab initio structure at the MP2/6-311G++(d,p) and B3LYP-D3(BJ)/6-311G++(d,p) levels.

MP2/6-311G++(d,p)			
Atom	X	Y	Z
N	0.423789	0.849665	-0.365476
C	1.211842	-0.217186	-1.005777
C	1.54865	1.315746	0.350869
C	-0.779516	0.439427	0.331735
S	0.829161	-1.702723	-0.031677
C	-0.909355	-1.11975	0.187827
C	-1.736534	-1.513338	-1.04106
C	-1.490902	-1.745709	1.454481
C	2.519907	0.53631	-0.564121
O	1.623507	1.97975	1.351794
N	3.635188	-0.090571	0.077443
C	-2.007866	1.142486	-0.218987
O	-3.025794	1.0691	0.67895
O	-2.101916	1.669503	-1.300132
H	1.024268	-0.358088	-2.07238
H	-0.676505	0.701167	1.392743
H	-1.699553	-2.598088	-1.172301
H	-1.360711	-1.038475	-1.952568
H	-2.784194	-1.218892	-0.907107
H	-1.556145	-2.833184	1.346449
H	-2.499322	-1.355804	1.625922
H	-0.868788	-1.513734	2.323946
H	2.870178	1.169403	-1.385802
H	4.212399	-0.603862	-0.579522
H	3.33588	-0.713986	0.820232
H	-3.780642	1.518716	0.268747

B3LYP-D3(BJ)/6-311G++(d,p)			
Atom	X	Y	Z
N	0.447341	0.814972	-0.345584
C	1.229163	-0.238782	-1.002237
C	1.558504	1.347748	0.321219
C	-0.774059	0.430013	0.329139
S	0.81069	-1.757382	-0.064845
C	-0.937698	-1.135215	0.193565
C	-1.790329	-1.530123	-1.018499
C	-1.500379	-1.744136	1.476627
C	2.541266	0.513356	-0.545391
O	1.628541	2.082227	1.264189
N	3.617852	-0.121302	0.146472
C	-1.985699	1.170308	-0.219913
O	-3.011461	1.11385	0.666395
O	-2.063771	1.718294	-1.284429
H	1.047763	-0.357091	-2.069459
H	-0.679032	0.690739	1.386323
H	-1.77902	-2.613829	-1.138114
H	-1.421984	-1.078033	-1.940566
H	-2.828579	-1.215423	-0.87694
H	-1.575429	-2.830013	1.386204
H	-2.502026	-1.349184	1.662437
H	-0.868018	-1.509058	2.334464
H	2.935031	1.10878	-1.372156
H	4.238991	-0.625958	-0.473254
H	3.295366	-0.736493	0.884571
H	-3.761541	1.583993	0.271505

Table S12. Cartesian coordinates in Angstroms of 6-APA, *Eq-Cis-IIb*, from the optimized ab initio structure at the MP2/6-311G++(d,p) and B3LYP-D3(BJ)/6-311G++(d,p) levels.

MP2/6-311G++(d,p)			
Atom	X	Y	Z
N	0.403324	-0.841255	0.342668
C	1.189985	0.219716	1.000448
C	1.538551	-1.311322	-0.356591
C	-0.778074	-0.410106	-0.385292
S	0.817448	1.71802	0.042953
C	-0.920379	1.138069	-0.182205
C	-1.742271	1.482034	1.065157
C	-1.523819	1.796085	-1.421115
C	2.500697	-0.533801	0.568994
O	1.624664	-1.975273	-1.356443
N	3.62118	0.094058	-0.063038
C	-2.061352	-1.115379	0.009712
O	-2.008159	-1.67049	1.237487
O	-3.043691	-1.137108	-0.700612
H	0.992784	0.348716	2.066997
H	-0.629886	-0.619753	-1.452334
H	-1.71558	2.562388	1.230992
H	-1.354543	0.983215	1.958384
H	-2.787878	1.181883	0.926777
H	-1.615592	2.876754	-1.272793
H	-2.52294	1.384823	-1.599665
H	-0.903468	1.609773	-2.302637
H	2.845176	-1.167607	1.392688
H	4.189841	0.611179	0.598428
H	3.327913	0.714837	-0.810479
H	-2.879048	-2.072962	1.380885

B3LYP-D3(BJ)/6-311G++(d,p)			
Atom	X	Y	Z
N	0.427511	-0.807127	0.317133
C	1.204455	0.239441	0.996811
C	1.550191	-1.337565	-0.334454
C	-0.77394	-0.400277	-0.387952
S	0.795281	1.772829	0.081582
C	-0.952078	1.152948	-0.184687
C	-1.796126	1.48757	1.051665
C	-1.539837	1.803607	-1.434609
C	2.521309	-0.5083	0.549348
O	1.634145	-2.067499	-1.279496
N	3.603945	0.132336	-0.127997
C	-2.039388	-1.144763	0.007379
O	-1.958306	-1.755976	1.203977
O	-3.031501	-1.158391	-0.67829
H	1.011637	0.341238	2.063989
H	-0.634123	-0.605265	-1.452216
H	-1.79726	2.565655	1.214492
H	-1.410448	1.005795	1.951592
H	-2.831246	1.165026	0.90626
H	-1.640838	2.882252	-1.295881
H	-2.532139	1.389131	-1.628252
H	-0.910354	1.62087	-2.307236
H	2.908575	-1.108104	1.376164
H	4.21472	0.638838	0.500539
H	3.287867	0.748136	-0.868412
H	-2.813299	-2.18634	1.359169

Table S13. Cartesian coordinates in Angstroms of 6-APA, *Ax-Cis-Ic*, from the optimized ab initio structure at the MP2/6-311G++(d,p) and B3LYP-D3(BJ)/6-311G++(d,p) levels.

MP2/6-311G++(d,p)			
Atom	X	Y	Z
N	-0.377627	1.03773	0.134953
C	-0.954552	0.23	1.224932
C	-1.614986	1.247586	-0.48409
C	0.81311	0.514719	-0.494577
S	-0.213052	-1.424488	1.088405
C	0.661076	-1.044444	-0.494272
C	-0.183198	-1.468483	-1.699126
C	1.998837	-1.786492	-0.494034
C	-2.378599	0.553482	0.663826
O	-1.911655	1.724181	-1.553008
N	-3.117565	-0.575672	0.130681
C	2.037299	0.980587	0.268893
O	3.107684	1.088727	-0.555741
O	2.071397	1.21069	1.454861
H	-0.743259	0.624522	2.220833
H	0.860289	0.878694	-1.526133
H	-0.355965	-2.548431	-1.662521
H	0.347217	-1.227448	-2.629068
H	-1.159898	-0.980304	-1.700956
H	1.820899	-2.866478	-0.492426
H	2.59898	-1.538641	0.385715
H	2.565441	-1.535036	-1.396823
H	-2.939875	1.231129	1.322532
H	-3.916169	-0.230658	-0.396349
H	-3.468662	-1.163494	0.882149
H	3.852856	1.363046	0.000926

B3LYP-D3(BJ)/6-311G++(d,p)			
Atom	X	Y	Z
N	-0.390594	1.007795	0.155596
C	-0.983954	0.191547	1.225922
C	-1.608428	1.286772	-0.45742
C	0.81114	0.510966	-0.473192
S	-0.245176	-1.4775	1.070193
C	0.680259	-1.058983	-0.504914
C	-0.141052	-1.473914	-1.729664
C	2.021522	-1.792892	-0.480158
C	-2.399123	0.544243	0.649669
O	-1.879417	1.826265	-1.495286
N	-3.12978	-0.561912	0.06603
C	2.03694	0.994855	0.284758
O	3.101067	1.121297	-0.542627
O	2.0829	1.231015	1.462125
H	-0.784159	0.56405	2.228503
H	0.85635	0.889643	-1.494826
H	-0.298709	-2.553919	-1.719514
H	0.394944	-1.210427	-2.647584
H	-1.122568	-1.001724	-1.739977
H	1.85504	-2.871932	-0.490589
H	2.602363	-1.550101	0.410636
H	2.607918	-1.533992	-1.365283
H	-2.97092	1.196987	1.321991
H	-3.950219	-0.225554	-0.426328
H	-3.42047	-1.23313	0.768564
H	3.855634	1.404735	-0.003773

Table S14. Cartesian coordinates in Angstroms of 6-APA, *Ax-Cis-IIc*, from the optimized ab initio structure at the MP2/6-311G++(d,p) and B3LYP-D3(BJ)/6-311G++(d,p) levels.

MP2/6-311G++(d,p)			
Atom	X	Y	Z
N	-0.362572	1.037926	0.079706
C	-0.918072	0.273812	1.212398
C	-1.614379	1.22192	-0.52172
C	0.809893	0.487178	-0.563301
S	-0.179124	-1.385148	1.132446
C	0.670178	-1.071279	-0.479023
C	-0.191406	-1.559553	-1.647065
C	2.016825	-1.795975	-0.469162
C	-2.353459	0.572954	0.667254
O	-1.933243	1.654807	-1.602342
N	-3.099257	-0.577521	0.193233
C	2.100083	0.997688	0.046532
O	1.983939	1.228436	1.37323
O	3.125073	1.152638	-0.578882
H	-0.689861	0.710521	2.187
H	0.811183	0.791475	-1.614951
H	-0.363425	-2.63589	-1.550713
H	0.325602	-1.368312	-2.595758
H	-1.168114	-1.070934	-1.660132
H	1.854482	-2.877757	-0.433659
H	2.623971	-1.512314	0.394493
H	2.57259	-1.562078	-1.383624
H	-2.903627	1.275097	1.3096
H	-3.905384	-0.255466	-0.336817
H	-3.438802	-1.133865	0.973366
H	2.856031	1.531537	1.669952

B3LYP-D3(BJ)/6-311G++(d,p)			
Atom	X	Y	Z
N	-0.376534	1.009852	0.08931
C	-0.942287	0.246622	1.213642
C	-1.612	1.253636	-0.506495
C	0.807398	0.481365	-0.551755
S	-0.194443	-1.422681	1.135431
C	0.688634	-1.088638	-0.484912
C	-0.162629	-1.579403	-1.660467
C	2.036907	-1.808358	-0.457825
C	-2.372217	0.561268	0.652653
O	-1.911102	1.740945	-1.561766
N	-3.10553	-0.577008	0.138918
C	2.096775	1.01512	0.050933
O	1.987801	1.29286	1.366402
O	3.117366	1.15779	-0.572618
H	-0.726571	0.675154	2.190645
H	0.80685	0.793098	-1.596496
H	-0.314998	-2.65724	-1.58114
H	0.349136	-1.37008	-2.605492
H	-1.146283	-1.111586	-1.673638
H	1.881317	-2.888359	-0.418434
H	2.638022	-1.520687	0.405315
H	2.601637	-1.579783	-1.364991
H	-2.935145	1.241527	1.305075
H	-3.93277	-0.270557	-0.361545
H	-3.385237	-1.210672	0.879694
H	2.853579	1.609757	1.665732

Table S15. Cartesian coordinates in Angstroms of 6-APA, *Ax-Trans-a*, from the optimized ab initio structure at the MP2/6-311G++(d,p) and B3LYP-D3(BJ)/6-311G++(d,p) levels.

MP2/6-311G++(d,p)			
Atom	X	Y	Z
N	-0.324062	1.03718	-0.032341
C	-0.949643	0.387752	1.14625
C	-1.559451	1.063088	-0.740643
C	0.882899	0.401173	-0.548885
S	-0.318049	-1.314208	1.191746
C	0.729917	-1.138689	-0.327772
C	2.030447	-1.906263	-0.080414
C	0.002782	-1.743197	-1.536083
C	-2.354503	0.667895	0.530892
O	-1.791542	1.251684	-1.906393
N	-3.288951	-0.424175	0.512143
C	2.141535	1.048425	0.050079
O	1.90885	2.107742	0.850765
O	3.255312	0.665509	-0.208089
H	-0.738447	0.876751	2.099922
H	0.923541	0.596058	-1.628432
H	1.793183	-2.967022	0.053809
H	2.556108	-1.550658	0.805981
H	2.696043	-1.805905	-0.941691
H	-0.23158	-2.794275	-1.342232
H	0.648775	-1.688324	-2.420918
H	-0.92974	-1.218603	-1.76008
H	-2.82116	1.544082	0.993844
H	-2.872814	-1.246883	0.084896
H	-4.122508	-0.173667	-0.011566
H	0.953631	2.284154	0.789111

B3LYP-D3(BJ)/6-311G++(d,p)			
Atom	X	Y	Z
N	-0.333366	1.011455	-0.000834
C	-0.965989	0.344744	1.162367
C	-1.555719	1.105777	-0.708109
C	0.884894	0.403529	-0.527352
S	-0.331733	-1.371473	1.173562
C	0.747223	-1.149931	-0.353178
C	2.047131	-1.917721	-0.108925
C	0.027792	-1.72739	-1.580221
C	-2.367481	0.649939	0.540619
O	-1.775402	1.35727	-1.857564
N	-3.294125	-0.441702	0.454125
C	2.138887	1.064304	0.073295
O	1.897975	2.144886	0.838197
O	3.252967	0.687272	-0.159639
H	-0.759457	0.808587	2.125295
H	0.925667	0.628089	-1.596923
H	1.818174	-2.980478	0.002715
H	2.566067	-1.578346	0.784551
H	2.721106	-1.799198	-0.958597
H	-0.207576	-2.78029	-1.417105
H	0.677933	-1.653803	-2.457832
H	-0.90017	-1.200759	-1.804582
H	-2.845467	1.500755	1.033063
H	-2.864038	-1.266716	0.049382
H	-4.117204	-0.193244	-0.082492
H	0.940364	2.315759	0.813558

Table S16. Cartesian coordinates in Angstroms of 6-APA, *Ax-Trans-b*, from the optimized ab initio structure at the MP2/6-311G++(d,p) and B3LYP-D3(BJ)/6-311G++(d,p) levels.

MP2/6-311G++(d,p)			
Atom	X	Y	Z
N	-0.318544	1.044932	-0.014619
C	-0.939824	0.383736	1.156912
C	-1.554036	1.075041	-0.730019
C	0.879572	0.403027	-0.545325
S	-0.289029	-1.307603	1.209874
C	0.71464	-1.137921	-0.33887
C	2.011508	-1.924459	-0.135986
C	-0.053296	-1.722717	-1.531497
C	-2.358759	0.648585	0.520058
O	-1.775934	1.304293	-1.887437
N	-3.32559	-0.387662	0.316364
C	2.14649	1.033471	0.054806
O	1.92483	2.09649	0.853121
O	3.256105	0.637344	-0.202016
H	-0.739689	0.877861	2.111487
H	0.915983	0.608301	-1.623269
H	1.76531	-2.984911	-0.016177
H	2.563562	-1.590687	0.742613
H	2.656715	-1.815311	-1.011654
H	-0.288649	-2.774315	-1.341821
H	0.567039	-1.662538	-2.434139
H	-0.988914	-1.191779	-1.725212
H	-2.837948	1.513988	0.99025
H	-3.896174	-0.527225	1.143399
H	-2.871888	-1.269315	0.092291
H	0.970268	2.279099	0.792219

B3LYP-D3(BJ)/6-311G++(d,p)			
Atom	X	Y	Z
N	-0.328027	1.015332	0.007822
C	-0.95926	0.343607	1.164628
C	-1.547634	1.105189	-0.712341
C	0.886545	0.403607	-0.522379
S	-0.324224	-1.368727	1.179374
C	0.741274	-1.151099	-0.357787
C	2.038192	-1.928723	-0.129949
C	0.006205	-1.718505	-1.58011
C	-2.373601	0.639366	0.517439
O	-1.751829	1.382947	-1.855309
N	-3.34033	-0.385592	0.283274
C	2.143264	1.055313	0.083169
O	1.905495	2.141188	0.841024
O	3.25588	0.668423	-0.141046
H	-0.759573	0.811412	2.128206
H	0.92936	0.633516	-1.590875
H	1.803765	-2.991066	-0.026128
H	2.567045	-1.599775	0.761478
H	2.705576	-1.806791	-0.984392
H	-0.229577	-2.771845	-1.420651
H	0.647437	-1.641447	-2.463907
H	-0.923191	-1.190013	-1.79316
H	-2.852766	1.491527	1.00523
H	-3.95462	-0.520134	1.076682
H	-2.901915	-1.272327	0.055627
H	0.948277	2.315402	0.810857

Table S17. Cartesian coordinates in Angstroms of 6-APA, *Ax-Trans-c*, from the optimized ab initio structure at the MP2/6-311G++(d,p) and B3LYP-D3(BJ)/6-311G++(d,p) levels.

MP2/6-311G++(d,p)			
Atom	X	Y	Z
N	-0.331545	1.038359	0.05654
C	-0.931824	0.307075	1.206136
C	-1.584927	1.178201	-0.588833
C	0.841439	0.421013	-0.554713
S	-0.157168	-1.325935	1.249849
C	0.674116	-1.12406	-0.390064
C	1.977317	-1.9244	-0.34375
C	-0.215711	-1.652874	-1.520022
C	-2.349591	0.563838	0.600711
O	-1.866658	1.565138	-1.692563
N	-3.069023	-0.617423	0.166352
C	2.135866	1.019198	0.016869
O	1.957297	2.019946	0.902834
O	3.230758	0.650002	-0.328353
H	-0.763808	0.797641	2.168961
H	0.82954	0.665247	-1.624575
H	1.733856	-2.987787	-0.248468
H	2.615482	-1.631806	0.490218
H	2.536224	-1.779632	-1.272296
H	-0.408917	-2.718569	-1.365109
H	0.298961	-1.525386	-2.48044
H	-1.183305	-1.149016	-1.555924
H	-2.92467	1.289627	1.193592
H	-3.898112	-0.33611	-0.351122
H	-3.36952	-1.167726	0.966635
H	1.001623	2.203296	0.907373

B3LYP-D3(BJ)/6-311G++(d,p)			
Atom	X	Y	Z
N	-0.340192	1.01361	0.08801
C	-0.957792	0.264309	1.213666
C	-1.575665	1.222477	-0.557888
C	0.843325	0.419792	-0.530439
S	-0.185172	-1.383562	1.233578
C	0.68956	-1.137744	-0.410317
C	1.994396	-1.932909	-0.354814
C	-0.187344	-1.649411	-1.559431
C	-2.366164	0.548355	0.589978
O	-1.832253	1.680847	-1.632312
N	-3.070646	-0.616237	0.097053
C	2.135217	1.033838	0.038058
O	1.948692	2.059497	0.88897
O	3.230865	0.668744	-0.285087
H	-0.804093	0.728446	2.187456
H	0.831987	0.689616	-1.589886
H	1.759922	-2.996715	-0.269625
H	2.62387	-1.643613	0.48342
H	2.564008	-1.781416	-1.273195
H	-0.362098	-2.719946	-1.440364
H	0.325585	-1.488154	-2.513036
H	-1.161588	-1.163898	-1.588613
H	-2.957615	1.24332	1.200342
H	-3.943299	-0.352171	-0.346313
H	-3.262344	-1.279673	0.839786
H	0.992655	2.234633	0.931889

Rotational spectrum analysis

The first step in the broadband spectrum analysis was removing the lines belonging to common species and photofragments found in previous studies.[9] The remaining spectrum has several lines with a complex, non-resolve hyperfine structure originated from the presence of two ^{14}N nuclei (I=1) in the molecule. The hyperfine structure occurs when a nucleus with non-homogenous nuclear change distribution ($I > 1/2$) interacts with the electric field gradient generated by the rest of the molecule. Consequently, there is a splitting of the rotation transitions in which the transition intensity is distributed among each hyperfine components. [10] A fact that reflects that this hyperfine structure belongs to 6-APA is that when the ablation laser pulse is blocked and hence does not hit the sample, the mentioned lines disappear from the spectrum. Because of the resolution of the LA-CP-FTMW spectrometer (100 kHz approximately), the hyperfine structure is not completely resolved as shown in Figure S03. Therefore, we only measured the frequency center of each transition when fitting each rotational line to a rigid rotor Hamiltonian.[11] Because the rotational frequencies were measured as the intensity-weighted mean of the central frequency, it affects the uncertainty of frequency measurements reflected in a slightly larger RMS value than the typical.

Guided by the spectroscopic parameters of each conformer collected in Table 1, the most stable conformer has a large dipole moment in the a-axis. Taking advantage of the characteristic pattern of a-type transitions we located *a-type-R-branch* progressions for the first rotamer (rotamer I) separated approximately $B+C\approx1017$ MHz. Then we obtained a first set of rotational constants that were used to make a more reliable prediction that allowed us to locate b- and c-type R-branch transitions that were added to the fit. 78 transitions were measured, determining the accurate rotational constants for this conformer collected in Table 1 in the main text.

Afterward, the lines belonging to rotamer I were subtracted from the spectrum, but many lines remained, suggesting the presence of more conformers. We located a new *a-type* progression separated ≈987 MHz and these transitions were added to the fit. Following the same procedure as that for the first conformer, *b-* and *c-type* transitions were located and added to the fit for a total of 68 transitions, resulting in a second set of accurate rotational constants (see Table 1 in the main text). Further spectrum exploration allowed us to obtain *b-* and *c-type R-branch* transitions, like those of rotamer I, but with no a-type transitions. The fit resulted in 43 transitions for this rotamer III. After this iterative process, *a-* and *b- type* transitions (54 transitions) were fitted for a fourth rotamer. Finally, a fifth rotamer was found with b-type transitions, very similar to those of rotamer IV, with additional *c-type* transitions, resulting in 31 transitions. The list of all the transitions measured for each of the different rotamers is compiled in Tables S03-S07.

We also qualitatively estimated the relative abundances of the axial and equatorial conformers by comparing the intensity of the rotational transitions (considering the whole intensity due to each hyperfine structure if resolved) in the broadband spectra and considering their predicted dipole moment components. A Boltzmann distribution at 298K was considered for the population estimation. The results show that the axial form is predominant and constitutes approximately 60% of the population. A comparison between the experimental results and the computational values shows that MP2 gives a better estimation on the energetics. This is reflected in the fact that we detect the five more stable structures according to MP2, while the same is not true for B3LYP where there is a switch in the energetical order. Additionally, the Eq-Trans-a conformer is observed with a higher intensity than predicted by B3LYP. Finally, according to B3LYP we should expect to observe the Ax-Cis-Ib conformer while the Eq-Cis Ia and

Ax-Cis-IIb should be either barely observable or both observed, and only Eq-Cis Ia is detected in good agreement with MP2.

Figure S03. $4_{2,4} \leftarrow 3_{2,1}$ and $4_{2,2} \leftarrow 3_{1,2}$ rotational transitions belonging to the Rotamer I and Rotamer II, respectively. *Top*: Non-resolved hyperfine structure using the LA-CP-FTMW spectrometer. *Center*: Completely resolved hyperfine structure employing the LA-MB-FTMW spectrometer. Each transition appears as a doublet due to the Doppler effect, and the resonance frequency is determined by the arithmetic mean of two Doppler components. The energy levels are labeled with the quantum numbers K_a, K_c, I, and F, and the quadrupole coupling Hamiltonian was set up in the coupled basis set (I_1, I_2, I, J, K, and F), where $I_1 + I_2 = I$, and $I + J = F$. *Bottom*: The corresponding predicted spectra before fitting.

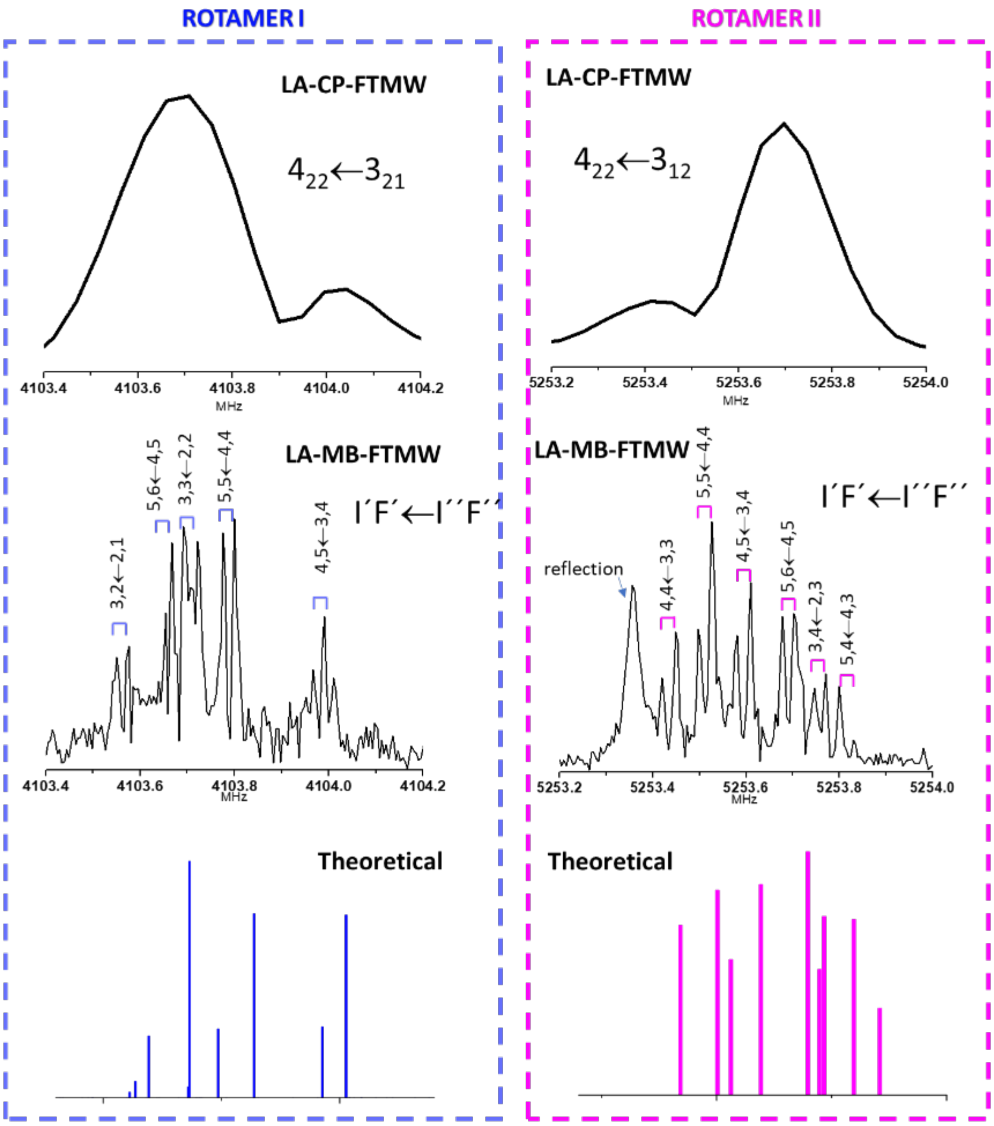

Table S18. Measured frequencies and residuals (in MHz) for the rotational transitions of rotamer I of 6-APA using the LA-CP-FTMW spectrometer. Note that, due to the nuclear quadrupole coupling, each line is split into several hyperfine components and only the center of frequencies was used.

J´	K´a	K´c	J´´	K´´a	K´´c	ν_{obs}	ν_{obs}-ν_{cal}
2	1	2	1	1	1	1988.670	-0.027
2	0	2	1	0	1	2032.009	-0.036
2	1	1	1	1	0	2082.756	0.058
3	1	3	2	1	2	2980.840	-0.005
3	0	3	2	0	2	3039.077	-0.006
3	2	2	2	2	1	3053.458	-0.088
3	1	2	2	1	1	3121.648	-0.021
3	1	3	2	0	2	3366.775	0.006
2	2	0	1	1	0	3374.250	0.086
2	2	1	1	1	1	3417.427	-0.086
2	2	0	1	1	1	3421.346	0.182
3	1	2	2	0	2	3648.882	0.289
4	1	4	3	1	3	3970.627	0.032
4	0	4	3	0	3	4036.085	-0.048
4	2	3	3	2	2	4068.659	0.108
4	3	2	3	3	1	4078.166	-0.029
4	3	1	3	3	0	4079.259	0.001
4	2	2	3	2	1	4103.688	-0.118
4	1	3	3	1	2	4157.489	-0.045
4	1	4	3	0	3	4298.379	0.099
3	2	2	2	1	1	4341.458	0.098
3	2	1	2	1	1	4359.599	0.123
3	2	2	2	1	2	4482.481	0.119
3	2	1	2	1	2	4500.612	0.135
5	0	5	4	1	4	4759.904	0.013
5	1	5	4	1	4	4957.554	0.032
5	0	5	4	0	4	5021.989	-0.050
6	1	5	5	2	3	5070.260	0.013
5	2	4	4	2	3	5081.046	-0.087
5	4	2	4	4	1	5097.390	0.035
5	3	3	4	3	2	5100.205	0.173
5	3	2	4	3	1	5103.721	-0.003
5	2	3	4	2	2	5148.188	-0.093
5	1	4	4	1	3	5188.755	-0.077
5	1	5	4	0	4	5219.792	0.123
4	2	3	3	1	2	5288.260	0.018
3	3	1	2	2	0	5315.790	-0.163
3	3	0	2	2	0	5316.199	0.068
3	3	0	2	2	1	5319.730	-0.053
4	2	2	3	1	2	5341.648	0.035
4	2	2	3	1	3	5623.547	0.110

6	0	6	5	1	5	5800.546	0.075
5	1	4	4	0	4	5919.708	-0.035
6	1	6	5	1	5	5941.412	-0.056
6	0	6	5	0	5	5998.155	0.052
6	2	5	5	2	4	6090.703	-0.026
6	5	2	5	5	1	6116.319	0.072
6	4	3	5	4	2	6119.381	-0.048
6	4	2	5	4	1	6119.789	0.103
6	3	4	5	3	3	6122.525	-0.037
6	3	3	5	3	2	6132.123	-0.132
6	1	6	5	0	5	6138.880	-0.219
6	2	4	5	2	3	6199.419	-0.043
6	1	5	5	1	4	6213.670	-0.105
4	3	2	3	2	1	6326.128	-0.010
4	3	1	3	2	1	6327.116	-0.264
5	2	3	4	1	3	6332.470	0.110
4	3	1	3	2	2	6345.477	-0.017
5	2	4	4	1	4	6680.553	-0.053
7	0	7	6	1	6	6826.587	-0.018
7	1	7	6	1	6	6922.529	-0.001
7	0	7	6	0	6	6967.646	0.044
7	1	7	6	0	6	7063.530	0.003
7	2	6	6	2	5	7096.799	-0.033
6	1	5	5	0	5	7111.507	0.027
7	5	3	6	5	2	7138.110	0.175
7	4	4	6	4	3	7142.781	-0.017
7	4	3	6	4	2	7143.612	-0.037
7	3	5	6	3	4	7145.174	-0.056
7	3	4	6	3	3	7166.408	-0.064
7	1	6	6	1	5	7230.320	0.042
4	4	1	3	3	0	7241.250	-0.013
4	4	0	3	3	1	7241.436	-0.013
7	2	5	6	2	4	7253.280	-0.040
5	3	2	4	2	3	7380.572	-0.096
8	0	8	7	1	7	7838.205	-0.005
8	1	8	7	1	7	7901.034	0.027
8	0	8	7	0	7	7934.212	0.076

Table S19. Measured frequencies and residuals (in MHz) for the rotational transitions of rotamer II of 6-APA using the LA-CP-FTMW spectrometer. Note that, due to the nuclear quadrupole coupling, each line is split into several hyperfine components and only the center of frequencies was used.

J′	K′a	K′c	J′′	K′′a	K′′c	ν_{obs}	ν_{obs}-ν_{cal}
3	2	2	2	1	1	4112.963	-0.087
5	0	5	4	1	4	4483.382	-0.100
3	2	2	2	1	2	4497.608	0.008
5	1	5	4	1	4	4528.963	0.009
5	0	5	4	0	4	4578.068	0.008
5	1	5	4	0	4	4623.561	0.028
3	2	1	2	1	2	4625.836	-0.160
5	2	4	4	2	3	4876.578	-0.005
4	2	3	3	1	2	4905.862	-0.065
5	4	1	4	4	0	4999.931	0.092
5	3	2	4	3	1	5067.851	0.156
4	1	3	3	0	3	5081.044	0.164
5	1	4	4	1	3	5099.985	-0.002
3	3	1	2	2	0	5189.355	0.088
3	3	0	2	2	0	5193.026	0.077
3	3	1	2	2	1	5216.433	0.056
3	3	0	2	2	1	5220.162	0.103
5	2	3	4	2	2	5231.738	-0.139
4	2	2	3	1	2	5253.694	0.061
6	0	6	5	1	5	5380.072	0.010
6	1	6	5	1	5	5400.387	0.016
6	0	6	5	0	5	5425.550	0.015
6	1	6	5	0	5	5445.860	0.017
6	1	5	5	2	4	5462.753	-0.022
5	2	4	4	1	3	5643.148	-0.064
4	2	3	3	1	3	5671.417	0.071
6	2	5	5	2	4	5805.473	-0.093
7	2	5	6	3	4	5862.058	0.218
6	3	4	5	3	3	5993.886	0.017
6	1	5	5	1	4	6006.015	0.015
6	4	2	5	4	1	6025.914	0.050
4	3	2	3	2	1	6120.403	0.163
4	3	1	3	2	1	6145.427	0.104
6	3	3	5	3	2	6156.956	0.028
4	3	2	3	2	2	6248.502	-0.134
7	0	7	6	1	6	6257.096	0.008
7	1	7	6	1	6	6265.726	0.006
7	0	7	6	0	6	6277.411	0.014
7	1	7	6	0	6	6286.046	0.017
6	2	4	5	2	3	6289.806	-0.092
7	1	6	6	2	5	6521.622	-0.083

7	2	6	6	2	5	6714.806	-0.033
7	1	6	6	1	5	6864.531	0.035
7	3	5	6	3	4	6975.566	-0.085
7	4	4	6	4	3	7030.019	-0.117
7	2	6	6	1	5	7057.665	0.035
7	4	3	6	4	2	7073.909	-0.087
4	4	1	3	3	0	7085.916	-0.053
4	4	0	3	3	0	7086.357	-0.025
4	4	1	3	3	1	7089.564	-0.086
4	4	0	3	3	1	7090.117	0.054
8	0	8	7	1	7	7124.179	0.006
8	1	8	7	1	7	7127.754	0.038
8	0	8	7	0	7	7132.822	0.017
8	1	8	7	0	7	7136.374	0.025
7	3	4	6	3	3	7271.881	-0.069
7	2	5	6	2	4	7304.731	-0.034
5	3	3	4	2	3	7318.449	-0.086
8	1	7	7	2	6	7507.551	-0.042
6	2	4	5	1	4	7536.132	0.009
8	2	7	7	2	6	7606.724	-0.002
8	1	7	7	1	6	7700.728	0.002
8	2	7	7	1	6	7799.940	0.080
8	3	6	7	3	5	7937.980	-0.090
9	0	9	8	1	8	7986.642	0.006
9	1	9	8	1	8	7988.061	0.008
9	0	9	8	0	8	7990.189	0.009
9	1	9	8	0	8	7991.615	0.019

Table S20. Measured frequencies and residuals (in MHz) for the rotational transitions of rotamer III of 6-APA using the LA-CP-FTMW spectrometer. Note that, due to the nuclear quadrupole coupling, each line is split into several hyperfine components and only the center of frequencies was used.

J′	K'_a	K'_c	J′′	K''_a	K''_c	ν_{obs}	ν_{obs}-ν_{cal}
2	2	1	1	1	0	3350.394	-0.173
2	2	0	1	1	0	3354.366	-0.120
3	1	3	2	0	2	3379.706	0.058
2	2	0	1	1	1	3402.670	0.188
3	1	2	2	0	2	3667.534	0.107
5	1	4	4	2	2	4092.512	-0.137
6	2	4	5	3	3	4157.957	0.118
7	3	4	6	4	3	4186.147	-0.043
4	1	4	3	0	3	4319.303	0.034
3	2	2	2	1	2	4474.142	0.202
4	1	3	3	0	3	4797.816	-0.019
5	0	5	4	1	4	4817.991	0.095
5	1	5	4	0	4	5249.264	0.069
3	3	1	2	2	0	5280.281	0.028
3	3	0	2	2	0	5280.579	0.125
3	3	1	2	2	1	5284.147	-0.024
3	3	0	2	2	1	5284.537	0.165
4	2	3	3	1	2	5284.866	0.025
6	1	5	5	2	4	5292.658	-0.084
4	2	2	3	1	2	5342.009	0.048
4	2	3	3	1	3	5572.773	0.154
6	0	6	5	1	5	5865.039	-0.048
5	1	4	4	0	4	5963.690	0.107
6	1	6	5	0	5	6177.855	-0.086
5	2	4	4	1	3	6215.914	-0.080
4	3	2	3	2	1	6299.385	-0.014
4	3	1	3	2	1	6300.801	0.002
4	3	2	3	2	2	6318.675	-0.146
4	3	1	3	2	2	6320.204	-0.018
5	2	3	4	1	3	6344.718	0.120
7	1	6	6	2	5	6441.604	-0.014
5	2	4	4	1	4	6694.424	-0.135
7	0	7	6	1	6	6897.231	0.014
7	1	7	6	0	6	7112.486	0.011
6	2	5	5	1	4	7125.167	0.005
6	1	5	5	0	5	7169.358	-0.046
4	4	1	3	3	0	7189.449	-0.111
4	4	1	3	3	1	7189.702	-0.059
5	3	3	4	2	2	7303.704	0.084
5	3	2	4	2	2	7309.171	-0.008
5	3	3	4	2	3	7360.558	-0.182

5	3	2	4	2	3	7366.264	-0.035
6	2	4	5	1	4	7368.848	0.037

Table S21. Measured frequencies and residuals (in MHz) for the rotational transitions of rotamer IV of 6-APA using the LA-CP-FTMW spectrometer. Note that, due to the nuclear quadrupole coupling, each line is split into several hyperfine components and only the center of frequencies was used.

J′	K'_a	K'_c	J′′	K''_a	K''_c	ν_{obs}	$\nu_{obs}-\nu_{cal}$
3	1	3	2	0	2	3054.576	0.034
2	2	1	1	1	0	3292.544	0.084
2	2	0	1	1	1	3423.973	0.100
4	0	4	3	1	3	3496.856	-0.054
4	0	4	3	0	3	3714.321	0.002
4	1	4	3	0	3	3843.394	-0.072
4	2	3	3	2	2	3867.754	0.129
5	1	4	4	2	3	4129.511	-0.083
3	2	2	2	1	1	4151.608	0.049
5	0	5	4	1	4	4438.980	-0.011
5	1	5	4	1	4	4508.099	0.014
5	0	5	4	0	4	4568.159	0.020
3	2	1	2	1	2	4581.622	0.111
5	1	5	4	0	4	4637.259	0.026
5	2	4	4	2	3	4810.146	0.021
5	2	3	4	2	2	5094.170	-0.054
3	3	1	2	2	0	5248.627	0.051
6	1	5	5	2	4	5264.656	0.049
3	3	0	2	2	1	5270.518	-0.019
6	0	6	5	1	5	5347.079	0.019
6	1	6	5	1	5	5381.397	0.013
6	0	6	5	0	5	5416.168	0.012
6	1	6	5	0	5	5450.489	0.011
5	2	4	4	1	3	5703.443	0.037
6	2	5	5	2	4	5737.613	-0.091
6	1	5	5	1	4	5945.149	0.012
6	3	3	5	3	2	5989.009	0.006
4	3	2	3	2	1	6177.859	0.008
7	0	7	6	1	6	6232.323	0.023
7	1	7	6	1	6	6248.504	0.002
7	0	7	6	0	6	6266.639	0.017
7	1	7	6	0	6	6282.836	0.010
4	3	1	3	2	2	6287.783	-0.268
7	1	6	6	2	5	6351.134	0.040
6	2	5	5	1	4	6418.193	-0.042
7	2	6	6	2	5	6649.524	0.007
7	1	6	6	1	5	6824.224	0.032
7	3	5	6	3	4	6855.424	0.028
5	3	3	4	2	2	7043.405	0.040
7	3	4	6	3	3	7060.481	-0.013
8	0	8	7	1	7	7104.401	0.010

8	1	8	7	1	7	7111.773	0.002
8	0	8	7	0	7	7120.605	0.009
7	2	6	6	1	5	7122.536	-0.079
8	1	8	7	0	7	7127.915	-0.059
7	2	5	6	2	4	7155.468	0.042
5	3	2	4	2	3	7365.676	-0.022
8	1	7	7	2	6	7374.038	-0.057
8	2	7	7	2	6	7546.306	0.002
8	1	7	7	1	6	7672.512	-0.005
8	2	7	7	1	6	7844.737	0.010
9	0	9	8	1	8	7969.611	0.005
9	1	9	8	1	8	7972.884	0.006
9	0	9	8	0	8	7976.979	-0.006

Table S22. Measured frequencies and residuals (in MHz) for the rotational transitions of rotamer V of 6-APA using the LA-CP-FTMW spectrometer. Note that, due to the nuclear quadrupole coupling, each line is split into several hyperfine components and only the center of frequencies was used.

J′	K'_a	K'_c	J′′	K''_a	K''_c	ν_{obs}	ν_{obs}-ν_{cal}
2	2	0	1	1	0	3289.342	-0.011
2	2	1	1	1	1	3383.925	-0.134
4	0	4	3	1	3	3532.248	-0.055
4	1	4	3	0	3	3843.955	-0.068
3	2	2	2	1	1	4129.510	0.080
3	2	1	2	1	1	4235.831	0.062
3	2	2	2	1	2	4480.241	-0.021
5	1	5	4	0	4	4643.624	0.016
4	2	3	3	1	2	4931.313	-0.001
4	1	3	3	0	3	4995.003	0.045
3	3	1	2	2	0	5206.892	0.111
3	3	0	2	2	0	5209.463	-0.018
4	2	2	3	1	2	5223.593	-0.115
3	3	1	2	2	1	5228.998	-0.020
6	0	6	5	1	5	5378.226	-0.027
6	1	6	5	0	5	5464.073	-0.005
4	2	3	3	1	3	5630.401	0.124
5	2	4	4	1	3	5680.803	0.047
4	3	2	3	2	1	6139.721	0.256
4	3	1	3	2	1	6158.067	0.096
4	3	2	3	2	2	6245.779	-0.025
7	0	7	6	1	6	6262.931	-0.003
7	1	7	6	0	6	6303.154	-0.016
7	1	6	6	2	5	6441.604	0.027
5	3	2	4	2	2	7073.414	-0.004
4	4	0	3	3	0	7109.010	-0.084
4	4	1	3	3	1	7111.427	-0.097
4	4	0	3	3	1	7111.778	-0.017
8	1	8	7	0	7	7153.905	-0.010
5	3	2	4	2	3	7365.623	-0.187
6	2	4	5	1	4	7426.991	0.053

Table S23. Measured frequencies and residuals (in MHz) for the nuclear quadrupole coupling hyperfine components of rotamer I of 6-APA using the LA-MB-FTMW spectrometer.

J′	K'_a	K'_c	I′	F′	J′′	K''_a	K''_c	I′′	F′′	ν_{obs}	ν_{obs}-ν_{cal}
4	1	4	3	4	3	1	3	2	3	3970.551	0.001
4	1	4	5	6	3	1	3	4	5	3970.565	0.003
4	1	4	5	4	3	1	3	4	3	3970.660	0.004
4	1	4	5	5	3	1	3	4	4	3970.699	0.008
4	2	3	5	6	3	2	2	4	5	4068.431	0.003
4	2	3	4	3	3	2	2	3	2	4068.523	0.004
4	2	3	4	5	3	2	2	3	4	4068.606	0.000
4	2	3	3	3	3	2	2	2	2	4068.680	-0.004
4	2	3	5	5	3	2	2	4	4	4068.724	0.006
4	2	3	4	4	3	2	2	3	3	4068.885	0.001
4	2	2	3	2	3	2	1	2	1	4103.563	0.001
4	2	2	5	6	3	2	1	4	5	4103.680	0.002
4	2	2	3	3	3	2	1	2	2	4103.710	-0.004
4	2	2	5	5	3	2	1	4	4	4103.788	0.000
4	2	2	4	5	3	2	1	3	4	4104.003	-0.001
3	2	2	3	4	2	1	1	2	3	4340.651	-0.003
3	2	2	3	3	2	1	1	2	2	4340.778	-0.001
3	2	2	4	4	2	1	1	3	4	4341.441	0.004
3	2	2	4	4	2	1	1	3	3	4341.881	0.001
3	2	2	2	2	2	1	1	1	2	4342.003	-0.003
3	2	2	2	2	2	1	1	1	1	4342.242	-0.002
3	2	1	4	5	2	1	1	3	4	4359.558	0.002
5	0	5	6	6	4	1	4	5	5	4759.616	0.000
5	0	5	4	5	4	1	4	3	4	4759.796	-0.001
5	0	5	6	7	4	1	4	5	6	4759.855	-0.001
5	0	5	4	5	4	1	4	4	5	4759.996	-0.001
5	0	5	5	6	4	1	4	4	5	4760.172	0.001
5	1	5	6	7	4	1	4	5	6	4957.520	0.004
5	1	5	6	5	4	1	4	5	4	4957.569	-0.005
5	0	5	5	6	4	0	4	4	5	5021.923	-0.001
5	0	5	6	7	4	0	4	5	6	5022.038	0.001
5	0	5	4	5	4	0	4	3	4	5022.064	-0.004
5	0	5	6	6	4	0	4	5	5	5022.208	0.003
5	2	3	6	7	4	2	2	5	6	5148.197	0.002
5	2	3	5	6	4	2	2	4	5	5148.412	-0.005
6	0	6	7	7	5	1	5	6	6	5800.290	-0.001
6	0	6	5	6	5	1	5	4	5	5800.427	0.002
6	0	6	7	8	5	1	5	6	7	5800.456	-0.002
6	0	6	6	7	5	1	5	5	6	5800.663	-0.001
6	1	6	6	7	5	1	5	5	6	5941.459	-0.002
6	1	6	7	8	5	1	5	6	7	5941.478	-0.005
6	1	6	7	7	5	1	5	6	6	5941.561	-0.002

Table S24. Measured frequencies and residuals (in MHz) for the nuclear quadrupole coupling hyperfine components of rotamer II of 6-APA using the LA-MB-FTMW spectrometer.

J′	K′a	K′c	I′	F′	J′′	K′′a	K′′c	I′′	F′′	ν_{obs}	ν_{obs}-ν_{cal}
5	1	5	6	6	4	0	4	5	5	4623.513	0.007
5	1	5	6	7	4	0	4	5	6	4623.548	0.002
5	2	4	6	5	4	2	3	5	4	4876.504	-0.001
5	2	4	6	7	4	2	3	5	6	4876.518	0.004
5	2	4	6	6	4	2	3	5	5	4876.640	0.001
5	2	4	5	5	4	2	3	4	4	4876.738	-0.001
4	2	3	5	5	3	1	2	4	4	4905.654	-0.002
4	2	3	3	3	3	1	2	2	2	4905.668	0.001
4	2	3	3	4	3	1	2	2	3	4906.037	-0.006
4	2	3	5	4	3	1	2	4	3	4906.188	0.002
3	3	0	3	3	2	2	0	2	2	5192.700	-0.004
3	3	0	4	4	2	2	0	2	3	5192.890	0.006
3	3	0	3	4	2	2	0	3	3	5192.934	-0.004
3	3	1	3	3	2	2	1	2	2	5216.154	0.000
3	3	1	4	4	2	2	1	3	3	5216.334	-0.006
3	3	1	3	4	2	2	1	2	3	5216.354	0.001
3	3	1	4	3	2	2	1	3	2	5216.432	-0.001
3	3	1	4	5	2	2	1	3	4	5216.500	0.000
4	2	2	4	4	3	1	2	3	3	5253.434	-0.003
4	2	2	5	5	3	1	2	4	4	5253.512	-0.002
4	2	2	4	5	3	1	2	3	4	5253.595	-0.001
4	2	2	5	6	3	1	2	4	5	5253.693	-0.001
4	2	2	3	4	3	1	2	2	3	5253.732	0.005
4	2	2	5	4	3	1	2	4	3	5253.786	-0.003
6	0	6	7	8	5	1	5	6	7	5380.045	-0.004
6	0	6	7	7	5	1	5	6	6	5380.079	0.003
5	2	4	6	6	4	1	3	5	5	5643.011	0.003
5	2	4	6	7	4	1	3	5	6	5643.304	0.004
5	2	4	5	6	4	1	3	4	5	5643.333	0.006
5	2	4	6	5	4	1	3	5	4	5643.392	-0.001
5	2	4	5	4	4	1	3	4	3	5643.421	-0.002
4	2	3	4	4	3	1	3	3	3	5671.086	0.001
4	2	3	5	5	3	1	3	3	4	5671.169	0.002
4	2	3	4	5	3	1	3	4	4	5671.326	0.004
4	2	3	5	6	3	1	3	4	5	5671.428	0.000
6	2	5	7	8	5	2	4	6	7	5805.527	0.001
6	2	5	5	5	5	2	4	4	4	5805.581	-0.002
6	2	5	6	7	5	2	4	5	6	5805.595	0.003
6	2	5	6	6	5	2	4	5	5	5805.649	-0.004
7	0	7	8	9	6	1	6	7	8	6257.079	-0.008
7	0	7	8	8	6	1	6	7	7	6257.093	-0.004
7	0	7	7	8	6	1	6	6	7	6257.108	0.002

Figure S04. Relaxed Potential Energy Scan of 6-APA calculated at B3LYP-D3(BJ)/6-311++G(d,p): (a) rotating the C-C-N-H dihedral angle. (b) NH_2 inversion performed through the transition state (qst3 method).

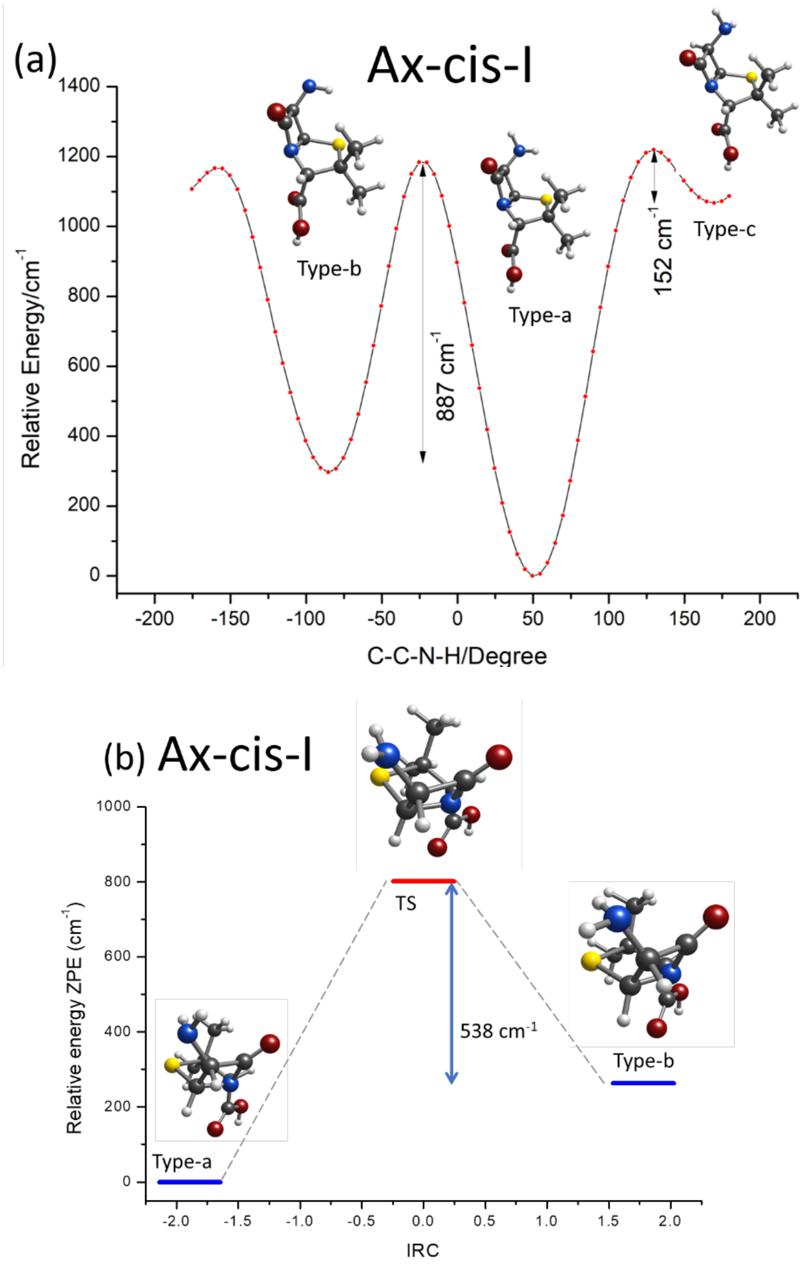

Figure S05. Comparison between the configurations *a, b* and *c*-type from an electrophilic attack.

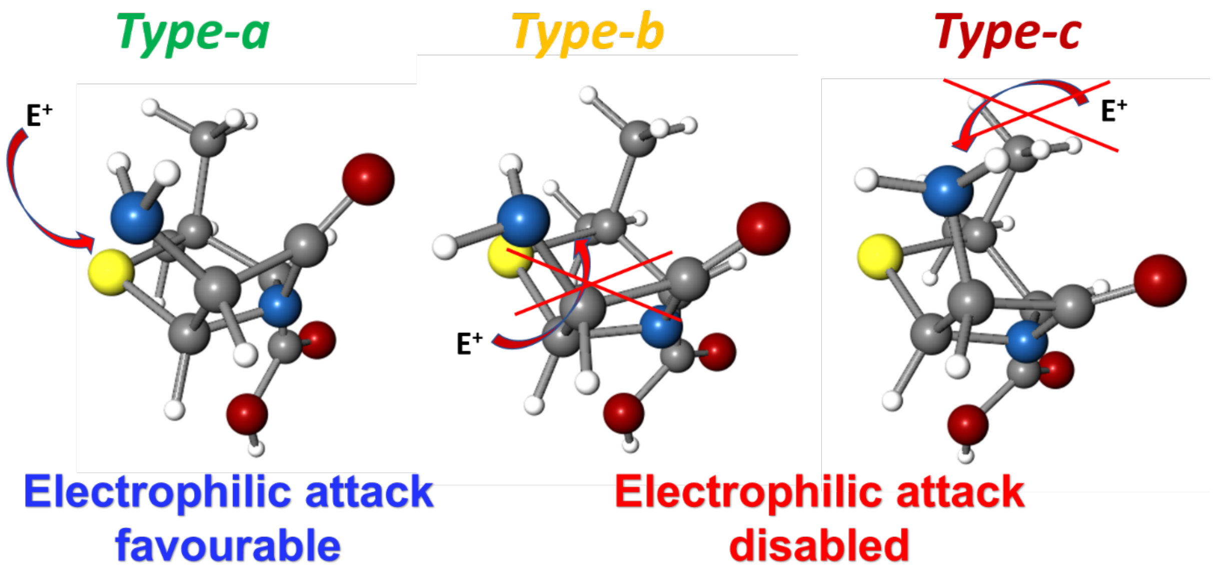

Figure S06. Comparison between the NCI plots of the *Ax-Cis-Ia* and *Eq-Trans-I* configurations of 6-APA. Red surfaces correspond to strong repulsion forces, blue to strong attraction forces, and green to weak attractive interactions. A value of 0.35 was used.

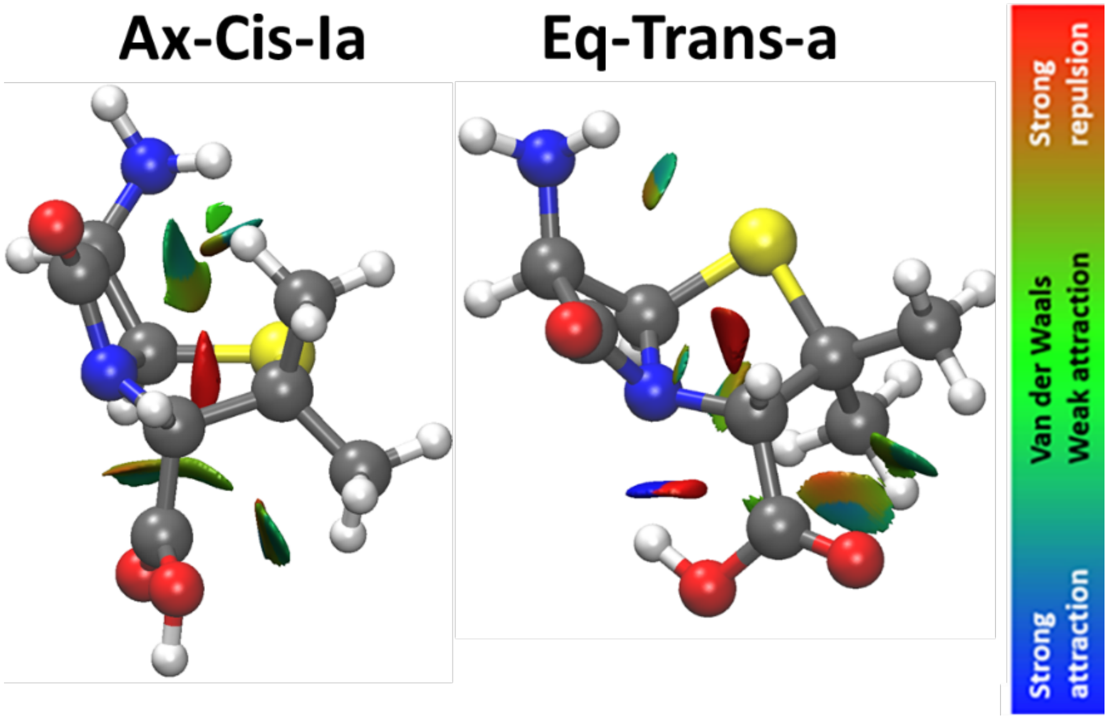

Figure S07. Atom numbering in the QTAIM analysis.

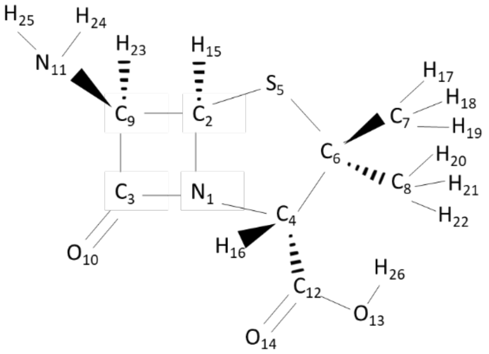

Table S25. Topological properties at the bond critical points (BCP) for the *Ax-Cis-Ia* conformer calculated using the QTAIM analysis at the MP2 method.

Ax-Cis-Ia							
Bond	ρ^a	$\nabla^2\rho^b$	V^c	G^d	V/G	H^e	ε^f
N_1-C_2	0.261	-0.653	-0.418	0.127	-3.284	-0.291	0.025
C_3-N_1	0.292	-0.803	-0.552	0.176	-3.143	-0.376	0.086
N_1-C_4	0.267	-0.711	-0.461	0.142	-3.254	-0.319	0.043
S_5-C_2	0.175	-0.251	-0.172	0.055	-3.151	-0.117	0.124
C_6-C_4	0.227	-0.467	-0.240	0.062	-3.897	-0.178	0.018
C_6-S_5	0.168	-0.223	-0.158	0.051	-3.093	-0.107	0.101
C_7-C_6	0.242	-0.552	-0.267	0.064	-4.148	0.202	0.005
C_6-C_8	0.241	-0.548	-0.267	0.065	-4.117	-0.202	0.013
C_9-C_2	0.236	-0.513	-0.255	0.063	-4.024	-0.192	0.028
C_3-C_9	0.242	-0.542	-0.269	0.067	-4.036	-0.202	0.059
O_{10}-C_3	0.404	0.073	-1.390	0.704	-1.974	-0.686	0.068
N_{11}-C_9	0.276	-0.774	-0.443	0.125	-3.553	-0.318	0.053
C_4-C_{12}	0.260	-0.659	-0.293	0.064	-4.559	-0.229	0.070
C_{12}-O_{13}	0.292	-0.392	-0.766	0.334	-2.293	-0.432	0.023
C_{12}-O_{14}	0.408	-0.002	-1.396	0.698	-2.001	-0.698	0.103
C_2-H_{15}	0.284	-0.990	-0.327	0.040	-8.200	-0.287	0.013
H_{16}-C_4	0.279	-0.956	-0.322	0.041	-7.778	-0.280	0.016
H_{17}-C_7	0.272	-0.909	-0.321	0.047	-6.835	-0.274	0.008
H_{18}-C_7	0.270	-0.895	-0.319	0.048	-6.705	-0.271	0.008
H_{19}-C_7	0.274	-0.921	-0.324	0.047	-6.931	-0.277	0.009
H_{22}-C_8	0.271	-0.904	-0.320	0.047	-6.799	-0.273	0.008
H_{20}-C_8	0.272	-0.906	-0.321	0.047	-6.790	-0.274	0.007
C_8-H_{21}	0.273	-0.917	-0.323	0.047	-6.918	-0.276	0.010
C_9-H_{23}	0.277	-0.945	-0.322	0.043	-7.484	-0.279	0.016
H_{24}-N_{11}	0.333	-1.578	-0.526	0.066	-7.999	-0.460	0.045
H_{25}-N_{11}	0.332	-1.546	-0.520	0.067	-7.787	-0.453	0.046
O_{13}-H_{26}	0.352	-2.479	-0.767	0.073	-10.432	-0.693	0.017
H_{19}-C_3	0.009	0.031	-0.005	0.007	-0.816	0.001	2.227

[a] Electron density at the BCP. [b] Laplacian at the BCP (au). [c] Potential energy density (au). [d] Gradient kinetic energy density (au). [e] Total electronic energy density (au). [f] Bond ellipticity.

Table S26. Topological properties at the bond critical points (BCP) for the *Eq-Trans-a* conformer calculated using the QTAIM analysis at the MP2 method.

Eq-Trans-a							
Bond	ρ^a	$\nabla^2\rho^b$	V^c	G^d	V/G	H^e	ε^f
C_2-N_1	0.250	-0.582	-0.378	0.116	-3.249	-0.262	0.010
C_3-N_1	0.284	-0.766	-0.492	0.150	-3.276	-0.342	0.069
N_1-C_4	0.254	-0.639	-0.423	0.132	-3.214	-0.291	0.030
S_5-C_2	0.180	-0.278	-0.184	0.057	-3.211	-0.127	0.093
C_6-C_4	0.232	-0.492	-0.248	0.063	-3.964	-0.186	0.017
S_5-C_6	0.170	-0.233	-0.162	0.052	-3.118	-0.110	0.089
C_6-C_7	0.242	-0.551	-0.267	0.064	-4.137	-0.202	0.004
C_8-C_6	0.243	-0.558	-0.271	0.066	-4.123	-0.205	0.012
C_9-C_2	0.237	-0.519	-0.259	0.065	-4.007	-0.194	0.034
C_9-C_3	0.244	-0.549	-0.272	0.068	-4.030	0.205	0.068
O_{10}-C_3	0.409	0.140	-1.429	0.732	-1.952	-0.697	0.070
N_{11}-C_9	0.278	-0.785	-0.447	0.125	-3.566	-0.321	0.049
C_4-C_{12}	0.252	-0.609	-0.280	0.064	-4.390	-0.216	0.089
C_{12}-O_{13}	0.299	-0.399	-0.792	0.346	-2.288	-0.446	0.010
C_{12}-O_{14}	0.409	-0.027	-1.399	0.696	-2.010	-0.703	-0.027
C_2-H_{15}	0.282	-0.972	-0.326	0.041	-7.886	-0.284	0.020
H_{16}-C_4	0.278	-0.944	-0.319	0.042	-7.647	-0.278	0.025
C_7-H_{17}	0.272	-0.908	-0.320	0.047	-6.875	-0.274	0.007
H_{18}-C_7	0.273	-0.914	-0.322	0.047	-6.891	-0.275	0.007
C_7-H_{19}	0.271	-0.900	-0.321	0.048	-6.670	-0.273	0.010
H_{22}-C_8	0.272	-0.911	-0.321	0.047	-6.878	-0.274	0.011
H_{20}-C_8	0.272	-0.906	-0.321	0.047	-6.798	-0.274	0.011
C_8-H_{21}	0.273	-0.918	-0.321	0.045	-7.045	-0.275	0.007
C_9-H_{23}	0.277	-0.945	-0.322	0.043	-7.486	-0.279	0.016
H_{24}-N_{11}	0.334	-1.590	-0.528	0.065	-8.090	-0.463	0.046
H_{25}-N_{11}	0.333	-1.566	-0.524	0.066	-7.894	-0.458	0.048
H_{26}-O_{13}	0.347	-2.450	-0.761	0.075	-10.217	-0.687	0.017
H_{21}-O_{14}	0.008	0.028	-0.005	0.006	-0.876	0.001	0.028
N_1-H_{26}	0.030	0.099	-0.025	0.025	-1.012	0.000	0.246

[a] Electron density at the BCP. [b]Laplacian at the BCP (au). [c]Potential energy density (au). [d]Gradient kinetic energy density (au). [e] Total electronic energy density (au). [f] Bond ellipticity.

Table S27. Nuclear charge calculated using the QTAIM analysis for conformers *Ax-Cis-Ia* and *Eq-Trans-a*.

Atom	Ax-Cis-Ia	Eq-Trans-a
N_1	-1.065	-1.056
C_2	0.220	0.179
C_3	1.373	1.353
C_4	0.359	0.302
S_5	0.006	0.030
C_6	-0.039	-0.032
C_7	0.004	-0.005
C_8	-0.002	-0.001
C_9	0.385	0.395
O_{10}	-1.139	-1.117
N_{11}	-1.043	-1.048
C_{12}	1.562	1.588
O_{13}	-1.128	-1.134
O_{14}	-1.132	-1.131
H_{15}	0.073	0.049
H_{16}	0.083	0.079
H_{17}	0.015	0.033
H_{18}	0.006	0.025
H_{19}	0.026	0.003
H_{20}	0.018	0.015
H_{21}	0.024	0.052
H_{22}	0.021	0.018
H_{23}	0.043	0.049
H_{24}	0.366	0.373
H_{25}	0.365	0.372
H_{26}	0.602	0.610

Figure S08. Comparison between the *Ax-Cis-Ia* structure and the structure observed in the crystal form of 6-APA.

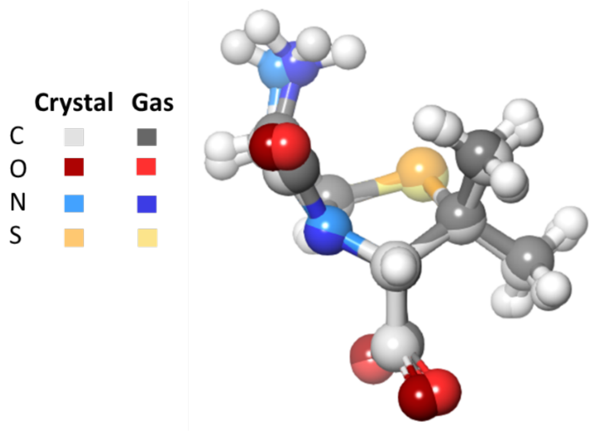

REFERENCES:

(1) Mata, S.; Pena, I.; Cabezas, C.; López, J. C.; Alonso, J. L. A Broadband Fourier-Transform Microwave Spectrometer with Laser Ablation Source: The Rotational Spectrum of Nicotinic Acid. *J. Mol. Spectrosc.* **2012**, *280*, 91–96. https://doi.org/http://dx.doi.org/10.1016/j.jms.2012.08.004.

(2) Peña, I.; Cabezas, C.; Alonso, J. L. The Nucleoside Uridine Isolated in the Gas Phase. *Angew. Chemie - Int. Ed.* **2015**, *54* (10), 2991–2994. https://doi.org/10.1002/anie.201412460.

(3) Schrödinger Release 2018-3: Maestro Schrödinger, LLC, New York, NY, 2018. Schrödinger Release 2018-3. Maestro. LLC: New York.

(4) Becke, A. D. Density-Functional Exchange-Energy Approximation with Correct Asymptotic Behavior. *Phys. Rev. A* **1988**, *38* (6), 3098. https://doi.org/10.1103/PhysRevA.38.3098.

(5) Becke, A. D. A New Mixing of Hartree-Fock and Local Density-Functional Theories. *J. Chem. Phys.* **1993**, *98* (2), 1372–1377. https://doi.org/10.1063/1.464304.

(6) Zhao, Y.; Truhlar, D. G. Density Functionals for Noncovalent Interaction Energies of Biological Importance. *J. Chem. Theory Comput.* **2007**, *3* (1), 289–300. https://doi.org/10.1021/ct6002719.

(7) Møller, C.; Plesset, M. S. Note on an Approximation Treatment for Many-Electron Systems. *Phys. Rev.* **1934**, *46* (7), 618. https://doi.org/10.1103/PhysRev.46.618.

(8) Frisch, M. J.; Pople, J. A.; Binkley, J. S. Self-Consistent Molecular Orbital Methods 25. Supplementary Functions for Gaussian Basis Sets. *J. Chem. Phys.* **1984**, *80* (7), 3265–3269. https://doi.org/10.1063/1.447079.

(9) Kolesniková, L.; León, I.; Alonso, E. R.; Mata, S.; Alonso, J. L. An Innovative Approach for the Generation of Species of the Interstellar Medium. *Angew. Chemie - Int. Ed.* **2021**, *60* (46), 24461–24466. https://doi.org/10.1002/ANIE.202110325.

(10) Gordy, W.; Cook, R. L. *Microwave Molecular Spectra*; Wiley: New York, 1984. https://doi.org/10.1016/0022-2860(72)80039-5.

(11) Pickett, H. M. The Fitting and Prediction of Vibration-Rotation Spectra with Spin Interactions. *J. Mol. Spectrosc.* **1991**, *148* (2), 371–377. https://doi.org/10.1016/0022-2852(91)90393-O.
